# Zirconocene catalyzed diastereoselective carbometalation of cyclobutenes†
†Electronic supplementary information (ESI) available: Experimental procedures, spectroscopic data and copies of ^1^H and ^13^C NMR spectra. See DOI: 10.1039/c6sc02617f



**DOI:** 10.1039/c6sc02617f

**Published:** 2016-08-30

**Authors:** Sudipta Raha Roy, Hendrik Eijsberg, Jeffrey Bruffaerts, Ilan Marek

**Affiliations:** a The Mallat Family Laboratory of Organic Chemistry , Schulich Faculty of Chemistry and Lise Meitner-Minerva Center for Computational Quantum Chemistry , Technion-Israel Institute of Technology , Technion City , Haifa 32000 , Israel . Email: chilanm@tx.technion.ac.il ; Fax: +972-4-829-37-09 ; Tel: +972-4-829-37-09

## Abstract

The regio- and diastereoselective zirconocene-catalyzed carbomagnesiation of cyclobutenes is herein reported to afford configurationally stable cyclobutylmagnesium species that could subsequently react with a large variety of electrophiles to give polysubstituted cyclobutane species as a single diastereoisomer.

## Abstract 2

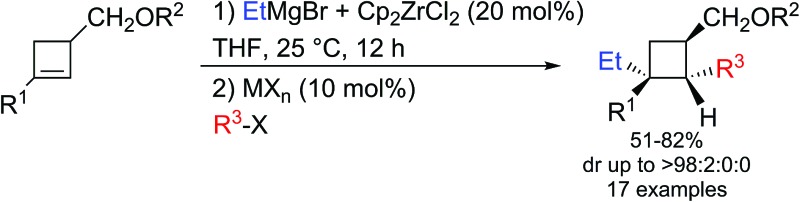
We functionalize cyclobutene species into polysubstituted metalated cyclobutanes through the Dzhemilev reaction.

## Introduction

**Scheme 1:**
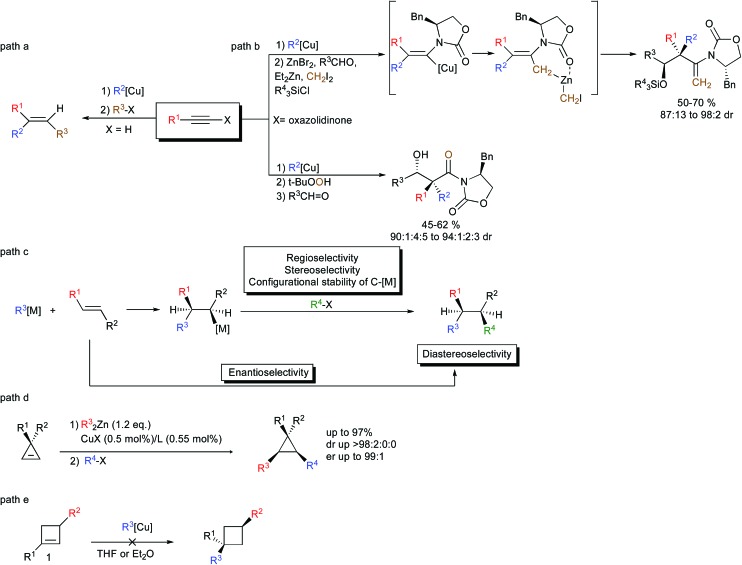
Carbometalation reactions.

In the repertoire of strategies using organometallic species that could lead to the efficient formation of two carbon–carbon bonds per chemical step, the carbometalation reaction to an unsaturated C–C bond represents a powerful strategy. The carbometalation reaction, defined as “the addition of a carbon–metal bond of an organometallic across a carbon–carbon unsaturated system leading to a new organometallic species that can be further functionalized” – is one of the most powerful approaches that have been used extensively to perform the 1,2-bis-alkylation of alkynes.^1^ In this context, organocopper,^2^ as well as zirconocene-catalyzed methylalumination,^3^ occupies a significant place due to its high stereoselectivity, typically controlled by the nature of the substituents on the triple bond (Scheme 1, path a for an example of carbocupration). Besides forming stereodefined polysubstituted double bonds, the carbometalation reaction of alkynes has recently been considered as a new stereodefined chemical handle to prepare reactive intermediates for the subsequent creation of more complex molecular structures possessing sp^3^-configurated stereocenters including quaternary carbon stereocenters (Scheme 1, path b).^4^ 1,2-Disubstituted alkyl chains possessing sp^3^ stereocenters could theoretically also be obtained through the carbometalation of appropriate alkenes (Scheme 1, path c).^5^ However, these transformations are much more challenging than the carbometalation reactions of alkynes, since the carbometalated product is usually of similar reactivity to the starting organometallic species and an oligomerization reaction typically occurs.^6^ Moreover, when the reaction is performed on the α,β-disubstituted double bond, several issues, such as: (1) regio- and stereoselectivity of the addition; (2) configurational stability of the resulting sp^3^ organometallic species; and (3) diastereoselectivity of the reaction with electrophiles; are of major concern.^7^ Finally, the enantioselectivity of the addition of a carbon nucleophile across an unactivated double bond still represents a very challenging problem despite the fact that it would acquire significant utility as a method for the creation of asymmetric vicinal carbon centers (Scheme 1, path c).^8^ Due to the inherent difficulty to achieve an efficient carbometalation reaction across unactivated alkenes, most of the studies have focused on strained double bonds. As such, the copper-mediated carbometalation reaction of cyclopropenyl derivatives^9^ has been investigated in detail to provide a new route to enantio- and diastereoenriched configurationally stable cyclopropyl metal species (Scheme 1, path d).^10^ However, all attempts to extend the concept of carbometalation to less-strained compounds such as cyclobutenes failed, most probably due to the lower energy release during the addition step.^11^ None of the copper-catalyzed carbomagnesiation, copper-catalyzed carbozincation, carbocupration with organocopper or organocuprate reactions in Et_2_O or THF could lead to the desired addition of the organometallic species across a double bond embedded in a 4-membered ring (Scheme 1, path e).^12^ Only Tortosa and coworkers recently reported the highly enantioselective desymmetrization of *meso*-cyclobutene through the copper-catalyzed borylation reaction.^13^

As stereodefined cyclobutyl metal species *en route* to polysubstituted cyclobutane derivatives still represent an important building block in the field of small ring chemistry,^14^ we therefore decided to pursuit our efforts to functionalize cyclobutene species into polysubstituted metalated cyclobutanes through carbometalation reaction, and more particularly through the Dzhemilev reaction. It should be emphasized that all starting cyclobutenes **1** were prepared by a rhodium-catalyzed intermolecular [2 + 2] cycloaddition of terminal alkynes with electron-deficient alkenes.^15^

## Results and discussion

**Scheme 2:**
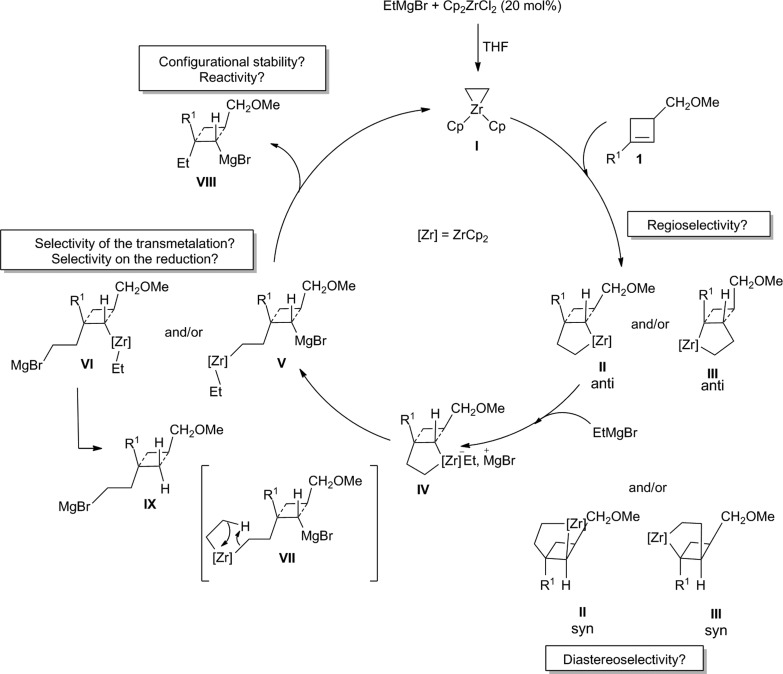
Proposed zirconocene-catalyzed diastereoselective ethylmagnesiation reaction of cyclobutene.

In this context, and as alluded previously, we were particularly interested in the possibility to reach our goal through the diastereoselective zirconocene-catalyzed carbomagnesiation reaction (Dzhemilev reaction) as described in Scheme 2.^16^

In this transformation, the addition of ethylmagnesium bromide to a catalytic amount of dichlorobis(η^5^-cyclopentadienyl)zirconium(iv) [Cp_2_ZrCl_2_] should provide the zirconacyclopropane **I** that would react *in situ* with the double bond of the cyclobutene **1** to form either the addition product **II** or **III**. Each of these two possible regioisomers could be present as potentially two diastereoisomers (**II** *anti versus* **II** *syn* and **III** *anti versus* **III** *syn*). So, not only the regioselectivity of the zirconocene-catalyzed carbomagnesiation of substituted cyclobutene **1** should be controlled (**II** *versus* **III**) but also the diastereoselectivity of the reaction (*syn versus anti*). Assuming that from the four possible regio- and diastereoisomers only the isomer **II** *anti* will be produced, the reaction of ethylmagnesium bromide with the zirconacyclopentane **II** *anti* would then provide the ate-complex **IV** that may either lead to the cyclobutyl magnesium **V** or cyclobutyl zirconocene species **VI** after transmetalation. The selectivity of the transmetalation is critical as the carbon attached to the zirconocene will then be subsequently reduced to regenerate the catalytic zirconacyclopropane species **I** (see for example the preparation of **VIII** *via* the reduction depicted in **VII**). If one assumes that the reaction would only provide **V**, then the cyclobutyl magnesium derivative **VIII** may be expected, whereas if the transmetalation occurs to provide **VI**, the cyclobutyl ethylmagnesium species **IX** is anticipated. Finally, if one still assumes that the catalytic cycle would only provide **VIII**, the configurational stability of this cyclobutyl magnesium species as well as its reactivity towards electrophiles needs to be investigated in detail.

**Scheme 3:**
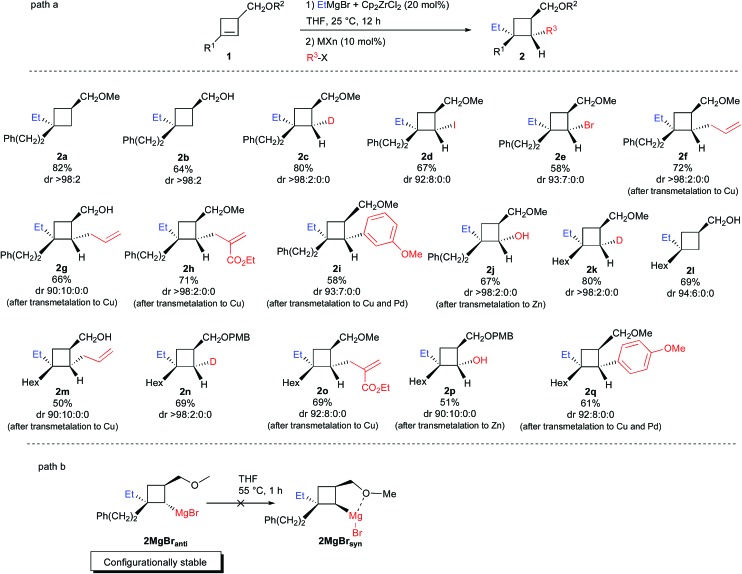
Zirconocene-catalyzed diastereoselective ethylmagnesiation reaction of cyclobutene and reaction with electrophiles.

Therefore, the Dzhemilev ethylmagnesiation of cyclobutene catalyzed by dichlorobis(η^5^-cyclopentadienyl)zirconium(iv) proceeds by a rather convoluted process with potentially several cyclic intermediates and the unique formation of **VIII**, requiring a complete control of all the elementary steps. We initially focused our attention on the diastereoselective zirconocene-mediated carbomagnesiation of cyclobutene **1a** (R^1^ = (CH_2_)_2_Ph, R^2^ = Me) in THF and we were pleased to observe that the addition reaction proceeds selectively under mild conditions (25 °C, 12 h) to provide **2a** in 82% isolated yield with >98 : 2 diastereoselectivity (Scheme 3). The relative configuration of cyclobutane **2a** was determined by the Nuclear Overhauser Effect (NOE) and from this analysis, we could confirm that the zirconocene-catalyzed ethylmagnesiation is not only highly regioselective (formation of **II** *versus* **III** in a 92 : 8 ratio) but also fully *anti*-diastereoselective (unique formation of **II** *anti versus* **II** *syn*, Scheme 2). As the reaction of a cyclobutene possessing an ether group (**1a**, R^1^ = (CH_2_)_2_Ph, R^2^ = Me) or an alcohol (**1b**, R^1^ = (CH_2_)_2_Ph, R^2^ = H) could potentially present a complementary sense of stereoinduction (reversal of stereoselectivity due to association of magnesium alkoxides with the zirconocene reagent),^17^ the same reaction was performed with **1b** and the product **2b** was obtained with the same diastereoisomeric ratio and with the same relative configuration albeit in slightly lower yield (64%, **2b** was then transformed into **2a** and the stereochemistry was corroborated).

Therefore, the uniform sense of stereoinduction of the reaction with **1a** and **1b** implies that the reaction is fully controlled by steric factors. Decreasing the basicity of the reaction medium and using Et_2_O as solvent instead of THF does not change the stereochemical outcome of the reaction and the major isomer **2a** was still observed with, however, higher quantity of product resulting from the formation of the opposite regioisomer **III** (**II** *anti* : **III** *syn* = 87 : 13, not shown in Scheme 3). Now that the regio- and diastereoselectivity of the zirconocene-catalyzed ethylmagnesiation reaction of cyclobutene **1a** is controlled, we were interested to understand the following step, namely the transmetalation step, and determine if cyclobutylmagnesium species **VIII** or its ethyl metalated cyclobutyl isomer **IX** would be formed. To answer this question, treatment of **1a** with ZrCp_2_Cl_2_ (20 mol%) and ethylmagnesium bromide was stirred at room temperature overnight and quenched with D_2_O/DCl. The cyclobutane **2c** was selectively obtained in 80% yield as a unique diastereoisomer (Scheme 3) suggesting complete selectivity in the transmetalation reaction. Only the cyclobutylmagnesium bromide **VIII** was therefore obtained through the reduction depicted in **VII**. Similarly, when the intermediate cyclobutylmagnesium species was trapped with I_2_ or NBS, **2d** and **2e** were isolated in 67% and in 58% yield, respectively, in an excellent diastereoisomeric ratio. The relative configuration of the cyclobutane **2d** was determined by NOE (see ESI†).

The unique stereochemistry of these functionalized cyclobutanes indicates that the cyclobutylmagnesium bond is configurationally stable at room temperature under this experimental condition. As the rate for inversion of configuration of the C–MgBr should be higher for cyclobutyl than for cyclopropyl, we were interested to check if the inversion of the organometallic species of **2MgBr*_anti_*** into **2MgBr*_syn_*** (Scheme 3, path b) could occur at higher temperature, as a chelated system would be preferentially formed. When the zirconocene-catalyzed ethylmagnesiation reaction was performed on **1a** (R^1^ = (CH_2_)_2_Ph, R^2^ = Me) at room temperature for 12 h in THF followed by warming at 55 °C for 1 h and finally quenching with I_2_, the same isomer **2d** was obtained with an identical diastereoisomeric ratio suggesting that the cyclobutylmagnesium bromide species is configurationally stable despite the potential stabilizing intramolecular chelation (Scheme 3, path b). The same configurational stability was observed when the isomerization was tested on **1d** (R^1^ = Hex, R^2^ = H).

Having a configurationally stable C–MgBr bond in the cyclobutylmagnesium bromide structure **VIII**, we were then concerned by the stereochemistry of transmetalation with copper salt. Would the resulting cyclobutylcopper species be produced with retention^18^ or inversion^19^ of configuration and would it also present some configurational stability? Thus, to the intermediate **VIII**, prepared as previously described from **1a** with ZrCp_2_Cl_2_ (20 mol%) and ethylmagnesium bromide, the corresponding cyclobutylcopper species was obtained by addition of CuI and LiCl (10 and 20 mol% respectively) at 0 °C for 15 min. Then, addition of allylbromide at 0 °C provided the allylated product **2f** in 72% yield with the same diastereoisomeric ratio of 98 : 2 : 0 : 0. The relative configuration of the cyclobutane **2f** was determined by NOE and indicates that the transmetalation reaction proceeds with retention of configuration to lead to a configurationally stable cyclobutyl carbon–copper bond.^20^ The transmetalation reaction was also performed on the intermediate resulting from the zirconocene-catalyzed carbomagnesiation reaction on alcohol **1b** (R^1^ = (CH_2_)_2_Ph, R^2^ = H) to check if a potential reversal of stereoselectivity with the copper salt may occur due to association with magnesium alkoxide. Further addition of allylbromide leads to the same major diastereoisomer **2g** in slightly lower ratio but still indicating that the transmetalation proceeds again with retention of configuration and that the C–Cu bond is configurationally stable. The reaction is not restricted to allylbromide and a functionalized electrophile could also be added successfully (formation of **2h**) with similar diastereoisomeric ratio and yield. For functionalization with a sp^2^-carbon center, an additional transmetalation to Pd is required and **2i** could be obtained in 58% yield and 93 : 7 : 0 : 0 diastereoisomeric ratio when the cyclobutylmagnesium species **VIII** is first transmetalated to copper salt [CuI (10 mol%) and LiCl (20 mol%) at 0 °C for 15 min] and then with Pd(Ph_3_)_4_ (10 mol%) followed by addition of 3-bromoanisole. Finally, due to the importance of diastereoisomerically pure cyclobutanol in synthesis,^21^ the simple oxidation of cyclobutyl zinc, easily obtained by transmetalation of **VIII** with ZnCl_2_ (1 equiv.) followed by addition of O_2_ gave the cyclobutanol **2j** in 67% yield and outstanding diastereoisomeric ratio.^22^ The zirconocene-catalyzed carbometalation reaction of cyclobutenes could be extended to various starting materials (R^1^ = PhCH_2_CH_2_; Hex; R^2^ = Me, H, PMB) and in all cases, the carbometalation remains highly diastereoselective and all subsequent transmetalation and reactions with electrophiles proceeded with retention of configuration (Scheme 3, formation of **2k** to **2q**). It should be noted that subsequent functionalization of cyclobutylmagnesium species **VIII** with an sp^2^-carbon center after transmetalation to Pd is not restricted to bromoarene, as **2q** could be obtained in 61% yield and in 92 : 8 : 0 : 0 diastereoisomeric ratio by addition of 4-iodoanisole.

## Conclusions

In conclusion, the zirconocene-catalyzed ethylmagnesiation of cyclobutene proceeds with a very high regio- and diastereoselectivity to give configurationally stable cyclobutylmagnesium species. The transmetalation reaction of the latter with copper and palladium salts proceeded with complete preservation of the stereochemical integrity. Reactions with various electrophiles led to different polysubstituted cyclobutyl derivatives in excellent yields and diastereoisomeric ratios.
