# Supplementary material for: Zirconocene catalyzed diastereoselective carbometalation of cyclobutenes
Source: Chem Sci. 2016 Aug 30;8(1):334–9. doi: 10.1039/c6sc02617f (PMC5365058; doi:10.1039/c6sc02617f)

Supplementary Information for

Zirconocene Catalyzed Diastereoselective Carbometalation of Cyclobutenes

Sudipta Raha Roy, Hendrik Eijsberg, Jeffery Bruffaerts and Ilan Marek*

Table of Contents:

**General Considerations:**	**1**
**Optimization of Reaction Conditions:**	**2**
**Experimental Section:**	**3**
Procedure for the synthesis of (3-hexylcyclobut-2-en-1-yl)methanol (**1b**):	**3**
Procedure for the synthesis of (3-phenethylcyclobut-2-en-1-yl)methanol (**1d**):	**3**
General procedure for the synthesis of cyclobutene methylethers (**1a/1c**):	**3**
Typical procedure for the synthesis of 1-(((3-hexylcyclobut-2-en-1-yl)methoxy)methyl)-4-methoxybenzene (**1e**):	**4**
General procedure for the synthesis of **2a, 2c, 2k,** and **2n**:	**4**
General procedure for the synthesis of **2b and 2l**:	**4**
Typical procedure for the synthesis of **2d**:	**5**
Typical procedure for the synthesis of **2e**:	**5**
Typical procedure for the synthesis of **2f**:	**5**
Typical procedure for the synthesis of **2g:**	**6**
General procedure for the synthesis of **2h** and **2o**:	**6**
Typical procedure for the synthesis of **2i**:	**6**
General procedure for the synthesis of **2j** and **2p**:	**7**
Typical procedure for the synthesis of **2m**:	**7**
Typical procedure for the synthesis of **2q**:	**7**
**Characterization of starting materials and compounds:**	**8-12**
**NMR spectra:**	**13-37**

General Considerations:

All glassware was flame dried under vacuum, and cooled under argon prior to use. Unless otherwise stated, all reactions were carried out under positive pressure of argon. Ether and THF were dried from Pure-Solv® Purification System (Innovative Technology©). Dichloromethane was distilled from CaH_2. Allyl bromide, 4-iodoanisole and 3-bromoanisole were distilled from $CaCl_2$. $[Rh(OMe)(1,5\text{-cod})]_2$ and anhydrous DMA was purchased from Aldrich and all other commercially obtained reagents were used as received. Ethylmagnesium bromide (2.5 M in diethyl ether) was prepared according to literature and freshly titrated before using with butanol/1,10-phenanthroline.[1] All organometallic compounds, dry solvents and reagents were transferred using plastic single-use graduated syringes and oven-dried stainless steel needles. Thin Layer Chromatography (TLC) was performed using Merck© silica gel 60 F254 plates and visualized by exposure to UV light (254 nm) or stained with anisaldehyde, phosphomolybdic acid, or potassium permanganate followed by heating. Column chromatography was performed using Bio-Lab silica gel 60A (0.040-0.063mm). Reactions were monitored and dr was determined from the crude reaction mixture by gas chromatography spectrometry (GC) using an Agilent Technologies 7820A GC with an Agilent Technologies 19091J-413 (30 m × 0.3 mm) column. Unless otherwise stated ^{1}H-NMR and ^{13}C-NMR spectra were recorded on a Bruker© spectrometers AVIII400, using $CDCl_3$ as a solvent. ^{1}H-^{1}H NOESY NMR experiments were performed in Bruker© spectrometers AVIII600. NMR data were processed with *Topspin* or with *NMRnotebook*. Peak multiplicities are reported as follows: s = singlet, d = doublet, t = triplet, dd = doublet of doublets, m= multiplet. High-resolution mass spectra (HRMS) were obtained by the mass spectrometry facility at the Technion.

1 A. B. Charette and H. Lebel, *Organic Syntheses*, 2004, Coll. Vol. *10*, 613.

Optimization of Reaction Conditions:

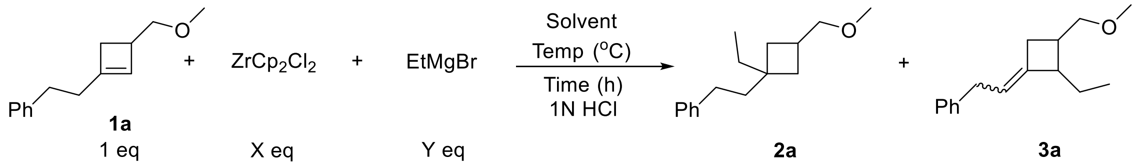

Entry[a]	X	Y	Solvent	Temp (^oC)	Time (h)	Conversion[b]	2a:3a[b]
1	1.5	5	Et_2O	rt	12	>99	87:13
2	1.5	5	THF	rt	4	>99	79:21
3	1.5	5	THF	-45 to rt	4	50	88:12
4	1.5	5	THF	-45 to rt	12	>99	93:7
5	1.1	3.5	THF	rt	4	57	81:19
6	1.1	3.5	THF	rt	12	57	81:19
**7**	**0.2**	**5**	**THF**	**rt**	**12**	**>99**	**92:8**[c]
8	0.2	5	Et_2O	rt	12	52	87:13
9	0.1	1.5	THF	rt	12	47	83:17

[a] $ZrCp_2Cl_2$ and EtMgBr were stirred for 30 min at rt in THF/Et_2O then **1a** was added and performed the reaction at varied condition. [b] Estimated by GC. [c] **3a** (dr ~8:2) was characterized by NMR and HRMS, see below.

Experimental section:

Synthesis of starting material:

Procedure for the synthesis of (3-phenethylcyclobut-2-en-1-yl)methanol (1b):
Following the reported procedure[2] rhodium pre-catalyst was synthesized from the commercially available $[Rh(OMe)(1,5\text{-}cod)]_2$. Butyl 3-phenethylcyclobut-2-ene-1-carboxylate was synthesized following the reported procedure[3] using this synthesized rhodium pre-catalyst from the reaction of 4-phenyl-but-1-yne and butyl acrylate. This crude butyl 3-phenethylcyclobut-2-ene-1-carboxylate (1 equiv, 10 mmol) was further reduced with $LiAlH_4$ (2 equiv, 20 mmol) in THF (40 mL) at 0 ^oC to synthesize title compound.
R_f = 0.50 (Hexane/EtOAc = 9:1); Yield: 53% (Pale yellow oil)
^1H NMR (400 MHz, $CDCl_3$): 2.01 (d, *J* = 13.2 Hz, 1H), 2.34 (t, *J* = 8.0 Hz, 2H), 2.49 (dd, *J* = 4.0, 13.2 Hz, 1H), 2.73 (t, *J* = 8.0 Hz, 2H), 2.83-2.87 (m, 1H), 3.61 (d, *J* = 6.0 Hz, 2H), 5.71 (s, 1H), 7.15-7.18 (m, 3H), 7.21-7.28 (2H); ^{13}C NMR (100 MHz, $CDCl_3$): δ = 32.9, 33.2, 34.1, 41.5, 66.1, 126.1, 128.1, 128.5, 128.5, 140.1, 151.5; HRMS (ESI) calcd. for $C_{13}H_{17}O$ $[M+H]^+$: 189.1276; found: 189.1278.

Procedure for the synthesis of (3-hexylcyclobut-2-en-1-yl)methanol (1d):
Following the reported procedure[2] rhodium pre-catalyst was synthesized from the commercially available $[Rh(OMe)(1,5\text{-}cod)]_2$. Butyl 3-hexylcyclobut-2-ene-1-carboxylate was synthesized following the reported procedure[3] using this synthesized rhodium pre-catalyst from the reaction of 1-octyne and butyl acrylate. This crude butyl 3-hexylcyclobut-2-ene-1-carboxylate (1 equiv, 10 mmol) was further reduced with $LiAlH_4$ (2 equiv, 20 mmol) in THF (40 mL) at 0 ^oC to synthesize title compound.
R_f = 0.53 (Hexane/EtOAc = 9:1); Yield: 51% (yellow oil)
^1H NMR (400 MHz, $CDCl_3$): δ = 0.85 (t, *J* = 7.2 Hz, 3H), 1.18-1.31 (m, 7H), 1.36-1.41 (m, 2H), 1.98 (t, *J* = 7.2 Hz, 2H), 2.07 (dd, *J* = 1.2, 13.2 Hz, 1H), 2.46 (dd, *J* = 4.0, 13.2 Hz, 1H), 2.81-2.83 (m, 1H), 3.62 (dd, *J* = 0.8, 6.0 Hz, 2H), 5.67 (d, *J* = 0.8 Hz, 1H); ^{13}C NMR (100 MHz, $CDCl_3$): δ = 14.3, 22.8, 26.8, 29.3, 31.3, 31.9, 34.0, 41.3, 66.4, 127.1, 152.6; HRMS (ESI) calcd. for $C_{11}H_{21}O$ $[M+H]^+$: 169.1592; found: 169.1590.

General procedure for the synthesis of cyclobutene methylethers (1a/1c):
To a flame dried 3-necked round bottom flask equipped with Teflon coated stirring bar, under argon atmosphere was added a suspension of NaH (1.5 equiv, 7.5 mmol) in THF (20 mL) at 0 ^oC. Then substituted cyclobutenol **1b**/**1d** (5 mmol, dissolved in 10 mL of THF) was added dropwise over a period of 2 min and the reaction mixture was allowed to stir at this temperature for an additional hour before introduction of MeI (1.2 equiv, 6 mmol). The reaction mixture was warmed to room temperature and stirred overnight. After that the reaction was completed, observed with TLC, the reaction mixture was cooled in an ice bath and quenched with water, extracted with Et_2O (3 times) and dried over $MgSO_4$. Combined organic layer was filtered and concentrated under reduced pressure to give crude oils which was then purified by flash chromatography using hexane/ Et_2O as eluent to get the desire **1a/1c** almost quantitative yield.

2 K. Sakai, T. Kochi and F. Kakiuchi, *Org. lett*., 2011, 13, 3928.
3 K. Sakai, T. Kochi and F. Kakiuchi, *Org. lett*., 2013, 15, 1024.

Typical procedure for the synthesis of 1-(((3-hexylcyclobut-2-en-1-yl)methoxy)methyl)-4-methoxybenzene (1e):

To a flame dried 3-necked round bottom flask equipped with Teflon coated stirring bar, under argon atmosphere was added a suspension of NaH (1.5 equiv, 3 mmol) in THF (8 mL) at 0 ^oC. Then substituted cyclobutenol (2 mmol, dissolved in 4 mL of THF) was added dropwise over a period of 2 min and the reaction mixture was allowed to stir at this temperature for an additional hour before introduction of 4-methoxy benzylbromide (1.2 equiv, 2.4 mmol) and tetra-*n*-butylammonium iodide (0.1 equiv, 0.2 mmol, dissolved in 2 mL of THF). The reaction mixture was warmed to room temperature and stirred overnight. After that the reaction was completed, observed by TLC, the reaction mixture was cooled in an ice bath and quenched with water, extracted with Et_2O (3 times) and dried over $MgSO_4$. Combined organic layer was filtered and concentrated under reduced pressure to give crude oils which was then purified by flash chromatography using hexane/ Et_2O as eluent to get the desire **1e** in 81% yield.

General procedure for the synthesis of 2a, 2c, 2k, and 2n:

Into a flame-dried, 25-mL three-neck flask, containing a solution of bis(cyclopentadienyl)zirconium dichloride (14.6 mg, 0.05 mmol) in dry THF (5 ml) and equipped with a magnetic stirrer, a glass stopper, a rubber septum and an inert gas inlet, was added a solution of EtMgBr (0.5 mL, 2.5 M in Et_2O, 1.25 mmol) at rt (22 °C) under inert atmosphere. After stirring for 30 min at rt, substrates **1a/1c/1e** (0.25 mmol), diluted in 2 ml of dry THF were added dropwise to this solution. The resulting mixture was allowed to stir at rt for overnight (12 h). During the course of reaction, the color of reaction mixture changes from pale yellow to dark yellow/ orange. The reaction mixture was then hydrolyzed (or deuterolyzed) with an aqueous solution of 1N HCl (or 1N DCl in D_2O). The layers were separated and the aqueous phase was extracted with Et_2O (3 times). The combined organic fractions were washed with brine, dried over $MgSO_4$, filtered and concentrated under reduced pressure to give crude oils. Finally, the crude mixtures were then purified by flash chromatography using hexane/ Et_2O as eluent.

General procedure for the synthesis of 2b and 2l:

Into a flame-dried, 25-mL three-neck flask, containing a solution of bis(cyclopentadienyl)zirconium dichloride (14.6 mg, 0.05 mmol) in dry THF (5 ml) and equipped with a magnetic stirrer, a glass stopper, a rubber septum and an inert gas inlet, was added a solution of EtMgBr (0.5 mL, 2.5 M in Et_2O, 1.25 mmol) at rt (22 °C) under inert atmosphere and stirring for 30 min at rt. In a parallel separate flask containing a solution of substrate **1b/1d** (0.25 mmol) in dry THF (2 ml) equipped with a magnetic stirrer, and a rubber septum was added a solution of EtMgBr (0.1 mL, 2.5 M in Et_2O, 0.25 mmol) under inert atmosphere at 0 °C and stirring for 30 min at the same temperature. Then the resulting substrate solution was transferred to the 25-mL three-neck flask through a cannula and the resulting mixture was allowed to stir at rt for overnight (12 h). During the course of reaction, the color of reaction mixture changes from pale yellow to dark yellow/ orange. The reaction mixture was then hydrolyzed with an aqueous solution of 1N HCl. The layers were separated and the aqueous phase was extracted with Et_2O (3 times). The combined organic fractions were washed with brine, dried over $MgSO_4$, filtered and concentrated under reduced pressure to give crude oils. Finally, the crude mixtures were then purified by flash chromatography using hexane/ Et_2O as eluent.

Typical procedure for the synthesis of 2d:

Into a flame-dried, 25-mL three-neck flask, containing a solution of bis(cyclopentadienyl)zirconium dichloride (14.6 mg, 0.05 mmol) in dry THF (5 ml) and equipped with a magnetic stirrer, a glass stopper, a rubber septum and an inert gas inlet, was added a solution of EtMgBr (0.5 mL, 2.5 M in Et_2O, 1.25 mmol) at rt (22 °C) under inert atmosphere. After stirring for 30 min at rt, substrates **1a** (0.25 mmol), diluted in 2 ml of dry THF were added dropwise to this solution. The resulting mixture was allowed to stir at rt for overnight (12 h). Then the electrophile, **iodine** (317 mg, 1.25 mmol) solubilized in dry THF (3 mL) was added at 0 °C and slowly warm-up to room temperature (over 2 h). Finally reaction mixture was hydrolyzed with an aqueous solution of 1N HCl. The layers were separated and the aqueous phase was extracted with Et_2O (3 times). The combined organic fractions were washed with saturated solution of sodium thiosulfate, dried over $MgSO_4$, filtered and concentrated under reduced pressure to give crude oil. Finally, the crude mixture was then purified by flash chromatography using hexane/ Et_2O as eluent to get **2d** in 67% yield.

Typical procedure for the synthesis of 2e:

Into a flame-dried, 25-mL three-neck flask, containing a solution of bis(cyclopentadienyl)zirconium dichloride (14.6 mg, 0.05 mmol) in dry THF (5 ml) and equipped with a magnetic stirrer, a glass stopper, a rubber septum and an inert gas inlet, was added a solution of EtMgBr (0.5 mL, 2.5 M in Et_2O, 1.25 mmol) at rt (22 °C) under inert atmosphere. After stirring for 30 min at rt, substrates **1a** (0.25 mmol), diluted in 2 ml of dry THF were added dropwise to this solution. The resulting mixture was allowed to stir at rt for overnight (12 h). Then for bromination, **NBS** (222 mg, 1.25 mmol) was solubilized in dry THF (4 mL) and added at 0 °C then slowly warm-up to room temperature (over 2 h). Finally reaction mixture was hydrolyzed with an aqueous solution of 1N HCl. The layers were separated and the aqueous phase was extracted with Et_2O (3 times). The combined organic fractions were washed with brime, dried over $MgSO_4$, filtered and concentrated under reduced pressure to give crude oil. Finally, the crude mixture was then purified by flash chromatography using hexane/ Et_2O as eluent to get **2e** in 58% yield.

Typical procedure for the synthesis of 2f:

Into a flame-dried, 25-mL three-neck flask, containing a solution of bis(cyclopentadienyl)zirconium dichloride (14.6 mg, 0.05 mmol) in dry THF (5 ml) and equipped with a magnetic stirrer, a glass stopper, a rubber septum and an inert gas inlet, was added a solution of EtMgBr (0.5 mL, 2.5 M in Et_2O, 1.25 mmol) at rt (22 °C) under inert atmosphere. After stirring for 30 min at rt, substrates **1a** (0.25 mmol), diluted in 2 ml of dry THF were added dropwise to this solution. The resulting mixture was allowed to stir at rt for overnight (12 h). Then CuI (4.8 mg, 0.025 mmol) and flame-dried LiCl (2.1 mg, 0.05 mmol) brought into solution in dry THF (3 mL) at 0 °C and wait for 15 min, followed by the addition of freshly distilled **allyl bromide** (108 µL,1.25 mmol) to the solution and stirred at rt for 3 h. Finally reaction mixture was hydrolyzed with an aqueous solution of 1N HCl. The layers were separated and the aqueous phase was extracted with Et_2O (3 times). The combined organic fractions were washed with brime, dried over $MgSO_4$, filtered and concentrated under reduced pressure to give crude oil which was then purified by flash chromatography using hexane/ Et_2O as eluent to get **2f** in 72% yield.

Typical procedure for the synthesis of 2g:

Into a flame-dried, 25-mL three-neck flask, containing a solution of bis(cyclopentadienyl)zirconium dichloride (14.6 mg, 0.05 mmol) in dry THF (5 ml) and equipped with a magnetic stirrer, a glass stopper, a rubber septum and an inert gas inlet, was added a solution of EtMgBr (0.5 mL, 2.5 M in Et_2O, 1.25 mmol) at rt (22 °C) under inert atmosphere and stirring for 30 min at rt. In a parallel separate flask containing a solution of substrate **1b** (0.25 mmol) in dry THF (2 ml) equipped with a magnetic stirrer, and a rubber septum was added a solution of EtMgBr (0.1 mL, 2.5 M in Et_2O, 0.25 mmol) under inert atmosphere at 0 °C and stirring for 30 min at the same temperature. Then the resulting substrate solution was transferred to the 25-mL three-neck flask through a cannula and the resulting mixture was allowed to stir at rt for overnight (12 h). Then CuI (4.8 mg, 0.025 mmol) and flame-dried LiCl (2.1 mg, 0.05 mmol) brought into solution in dry THF (3 mL) at 0 °C and wait for 15 min, followed by the addition of freshly distilled **allyl bromide** (130 µL,1.5 mmol) to the solution and stirred at rt for 3 h. Finally reaction mixture was hydrolyzed with an aqueous solution of 1N HCl. The layers were separated and the aqueous phase was extracted with Et_2O (3 times). The combined organic fractions were washed with brime, dried over $MgSO_4$, filtered and concentrated under reduced pressure to give crude oil which was then purified by flash chromatography using hexane/ Et_2O as eluent to get **2g** in 66% yield.

General procedure for the synthesis of 2h and 2o:

Into a flame-dried, 25-mL three-neck flask, containing a solution of bis(cyclopentadienyl)zirconium dichloride (14.6 mg, 0.05 mmol) in dry THF (5 ml) and equipped with a magnetic stirrer, a glass stopper, a rubber septum and an inert gas inlet, was added a solution of EtMgBr (0.5 mL, 2.5 M in Et_2O, 1.25 mmol) at rt (22 °C) under inert atmosphere. After stirring for 30 min at rt, substrates **1a/1c** (0.25 mmol), diluted in 2 ml of dry THF were added dropwise to this solution. The resulting mixture was allowed to stir at rt for overnight (12 h). Then CuI (4.8 mg, 0.025 mmol) and flame-dried LiCl (2.1 mg, 0.05 mmol) brought into solution in dry THF (3 mL) at 0 °C and wait for 15 min, followed by the addition of freshly distilled **ethyl 2-(bromomethyl)acrylate** (241 mg,1.25 mmol) to the solution and stirred at rt for 3 h. Finally reaction mixture was hydrolyzed with an aqueous solution of 1N HCl. The layers were separated and the aqueous phase was extracted with Et_2O (3 times). The combined organic fractions were washed with brime, dried over $MgSO_4$, filtered and concentrated under reduced pressure to give crude oil. Finally, the crude mixture was then purified by flash chromatography using hexane/ Et_2O as eluent.

Typical procedure for the synthesis of 2i:

Into a flame-dried, 25-mL three-neck flask, containing a solution of bis(cyclopentadienyl)zirconium dichloride (14.6 mg, 0.05 mmol) in dry THF (5 ml) and equipped with a magnetic stirrer, a glass stopper, a rubber septum and an inert gas inlet, was added a solution of EtMgBr (0.5 mL, 2.5 M in Et_2O, 1.25 mmol) at rt (22 °C) under inert atmosphere. After stirring for 30 min at rt, substrates **1a** (0.25 mmol), diluted in 2 ml of dry THF were added dropwise to this solution. The resulting mixture was allowed to stir at rt for overnight (12 h). Then CuI (4.8 mg, 0.025 mmol) and flame-dried LiCl (2.1 mg, 0.05 mmol) brought into solution in dry THF (3 mL) at 0 °C and wait for 15 min, followed by the addition of 3-bromoanisole (234 mg, 1.25 mmol) and $Pd(PPh_3)_4$ (28.9 mg, 0.025 mmol) solubilized in dry THF (3 mL) respectively at rt. Then resulting mixture was allowed to stir at 50 °C for 5 hours at which time it was quenched with an aqueous solution of 1N HCl. The layers were separated and the aqueous phase was extracted with Et_2O (3 times). The combined organic fractions were washed with brime, dried over $MgSO_4$, filtered and concentrated under reduced pressure to give crude oil. Finally, the crude mixture was then purified by flash chromatography using hexane/ Et_2O as eluent to get **2i** in 58% yield.

General procedure for the synthesis of 2j and 2p:

Into a flame-dried, 25-mL three-neck flask, containing a solution of bis(cyclopentadienyl)zirconium dichloride (14.6 mg, 0.05 mmol) in dry THF (5 ml) and equipped with a magnetic stirrer, a glass stopper, a rubber septum and an inert gas inlet, was added a solution of EtMgBr (0.5 mL, 2.5 M in Et_2O, 1.25 mmol) at rt (22 °C) under inert atmosphere. After stirring for 30 min at rt, substrates **1a/1e** (0.25 mmol), diluted in 2 ml of dry THF were added dropwise to this solution. The resulting mixture was allowed to stir at rt for overnight (12 h). Then freshly prepared $ZnCl_2$ solution in THF (0.25 mL, 1 M, 0.25 mmol) was added at 0 ^oC and stirred for 15 min, followed by the slow bubbling of $\mathbf{O_2}$ from a balloon for 1 h. Finally reaction mixture was hydrolyzed with an aqueous solution of 1N HCl. The layers were separated and the aqueous phase was extracted with Et_2O (3 times). The combined organic fractions were washed with brime, dried over $MgSO_4$, filtered and concentrated under reduced pressure to give crude oil. Finally, the crude mixture was then purified by flash chromatography using hexane/ EtOAc as eluent.

Typical procedure for the synthesis of 2m:

Into a flame-dried, 25-mL three-neck flask, containing a solution of bis(cyclopentadienyl)zirconium dichloride (87.6 mg, 0.3 mmol) in dry THF (5 ml) and equipped with a magnetic stirrer, a glass stopper, a rubber septum and an inert gas inlet, was added a solution of EtMgBr (1.0 mL, 2.5 M in Et_2O, 2.5 mmol) at rt (22 °C) under inert atmosphere. After stirring for 30 min at rt, substrates **1d** (0.25 mmol), diluted in 2 ml of dry THF were added dropwise to this solution. The resulting mixture was allowed to stir at rt for overnight (12 h). Then CuI (4.8 mg, 0.025 mmol) and flame-dried LiCl (2.1 mg, 0.05 mmol) brought into solution in dry THF (3 mL) at 0 °C and wait for 15 min, followed by the addition of freshly distilled **allyl bromide** (216 μL, 2.5 mmol) to the solution and stirred at rt for 3 h. Finally reaction mixture was hydrolyzed with an aqueous solution of 1N HCl. The layers were separated and the aqueous phase was extracted with Et_2O (3 times). The combined organic fractions were washed with brime, dried over $MgSO_4$, filtered and concentrated under reduced pressure to give crude oil which was then purified by flash chromatography using hexane/ Et_2O as eluent to get **2m** in 50% yield.

Typical procedure for the synthesis of 2q:

Into a flame-dried, 25-mL three-neck flask, containing a solution of bis(cyclopentadienyl)zirconium dichloride (14.6 mg, 0.05 mmol) in dry THF (5 ml) and equipped with a magnetic stirrer, a glass stopper, a rubber septum and an inert gas inlet, was added a solution of EtMgBr (0.5 mL, 2.5 M in Et_2O, 1.25 mmol) at rt (22 °C) under inert atmosphere. After stirring for 30 min at rt, substrates **1c** (0.25 mmol), diluted in 2 ml of dry THF were added dropwise to this solution. The resulting mixture was allowed to stir at rt for overnight (12 h). Then CuI (4.8 mg, 0.025 mmol) and flame-dried LiCl (2.1 mg, 0.05 mmol) brought into solution in dry THF (3 mL) at 0 °C and wait for 15 min, followed by the addition of **4-iodoanisole** (293 mg, 1.25 mmol) and $Pd(PPh_3)_4$ (28.9 mg, 0.025 mmol) solubilized in dry THF (3 mL) respectively at rt. Then resulting mixture was allowed to stir at 50 °C for 4 hours at which time it was quenched with an aqueous solution of 1N HCl. The layers were separated and the aqueous phase was extracted with Et_2O (3 times). The combined organic fractions were washed with brime, dried over $MgSO_4$, filtered and concentrated under reduced pressure to give crude oil. Finally, the crude mixture was then purified by flash chromatography using hexane/ Et_2O as eluent to get **2q** in 61% yield.

Characterization of starting materials:

(2-(3-(Methoxymethyl)cyclobut-1-en-1-yl)ethyl)benzene (1a):

R_f = 0.47 (Hexane/Et_2O = 98:2); Yield: 95% (Pale yellow oil)

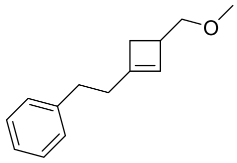

^1H NMR (400 MHz, $CDCl_3$): 2.00 (dd, *J* = 0.8, 13.2 Hz, 1H), 2.26 (t, *J* = 8.0 Hz, 2H), 2.47 (dd, *J* = 4.0, 13.2 Hz, 1H), 2.68 (t, *J* = 8.0 Hz, 2H), 2.79-2.81 (m, 1H), 3.28-3.35 (m, 5H), 5.70 (d, *J* = 0.8 1H), 7.08-7.12 (m, 3H), 7.18-7.22 (2H); ^{13}C NMR (100 MHz, $CDCl_3$): δ = 32.8, 33.0, 34.8, 38.9, 58.9, 76.9, 125.8, 128.3 (x2), 128.9, 142.0, 150.2; HRMS (ESI) calcd. for $C_{14}H_{19}O$ $[M+H]^+$: 203.1436; found: 203.1440.

1-Hexyl-3-(methoxymethyl)cyclobut-1-ene (1c):

R_f = 0.53 (Hexane/Et_2O = 98:2); Yield: 93% (yellow oil)

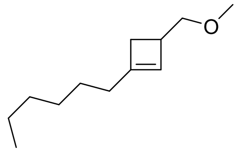

^1H NMR (400 MHz, $CDCl_3$): δ = 0.87 (t, *J* = 7.2 Hz, 3H), 1.27-1.32 (m, 7H), 1.37-1.42 (m, 2H), 1.97-2.04 (m, 2H), 2.52 (dd, *J* = 4.0, 13.2 Hz, 1H), 2.83-2.85 (m, 1H), 3.34-3.42 (m, 5H), 5.72 (s, 1H); ^{13}C NMR (100 MHz, $CDCl_3$): δ = 14.1, 22.6, 26.6, 29.1, 31.0, 31.7, 34.7, 38.8, 58.8, 77.1, 128.0, 151.3; HRMS (ESI) calcd. for $C_{12}H_{23}O$ $[M+H]^+$: 183.1749; found: 183.1752.

1-Hexyl-3-(methoxymethyl)cyclobut-1-ene (1e):

R_f = 0.51 (Hexane/Et_2O = 98:2); Yield: 81% (Colorless oil)

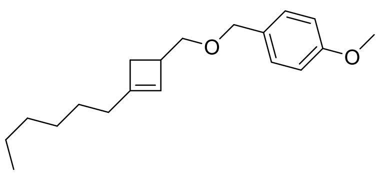

^1H NMR (400 MHz, $CDCl_3$): δ = 0.81 (t, *J* = 7.2 Hz, 3H), 1.19-1.24 (m, 6H), 1.29-1.36 (m, 2H), 1.89-1.96 (m, 3H), 2.45 (dd, *J* = 4.0, 12.8 Hz, 1H), 2.80-2.81 (m, 1H), 3.32-3.34 (m, 2H), 3.73 (s, 3H), 4.38 (s, 2H), 5.65 (d, *J* = 0.8 Hz, 1H), 6.80 (d, *J* = 8.4 Hz, 2H), 7.18 (d, *J* = 8.4 Hz, 2H); ^{13}C NMR (100 MHz, $CDCl_3$): δ = 14.1, 22.6, 26.6, 29.1, 31.0, 31.7, 34.8, 38.9, 55.3, 72.7, 74.4, 113.7, 128.2, 129.2, 130.8, 151.2, 159.1; HRMS (ESI) calcd. for $C_{19}H_{29}O_2$ $[M+H]^+$: 289.2168; found: 289.2175.

Characterization of compounds:

(2-(1-Ethyl-3-(methoxymethyl)cyclobutyl)ethyl)benzene (2a):

R_f = 0.50 (Hexane/Et_2O = 98:2); dr = >98:2; Yield: 82% (Colorless oil)

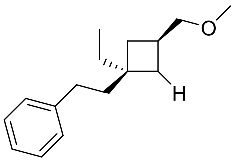

^1H NMR of major isomer (400 MHz, $CDCl_3$):0.79 (t, *J* = 7.6 Hz, 3H), 1.39-1.44 (m, 2H), 1.48-1.60 (m, 4H), 1.77-1.83 (m, 2H), 2.34-2.42 (m, 3H), 3.26-3.28(m, 5H), 7.07-7.11 (m, 3H), 7.18-7.22 (m, 2H); ^{13}C NMR of major isomer (100 MHz, $CDCl_3$): δ = 8.3, 28.2, 29.8, 30.2, 34.9 (x2), 38.1, 42.0, 58.8, 78.7, 125.5, 128.3, 128.3, 143.2; HRMS (ESI) calcd. for $C_{16}H_{25}O$ $[M+H]^+$: 233.1900; found: 233.1888.

(3-Ethyl-3-phenethylcyclobutyl)methanol (2b):

R_f = 0.51 (Hexane/EtOAc = 8:2); dr = >98:2; Yield: 64% (Colorless oil)

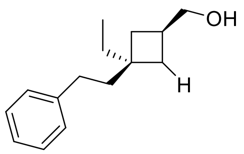

^1H NMR of major isomer (400 MHz, $CDCl_3$): δ = 0.85 (t, *J* = 7.2 Hz, 3H), 1.44-1.49 (m, 2H), 1.55-1.66 (m, 5H), 1.81-1.86 (m, 2H), 2.38-2.44 (m, 3H), 3.57 (d, *J* = 6.8 Hz, 2H), 7.13-7.17 (m, 3H), 7.24-7.27 (m, 2H) ; ^{13}C NMR of major isomer (100 MHz, $CDCl_3$): δ = 8.3, 29.9, 30.2, 30.5, 34.2 (x2), 37.9, 41.9, 68.3, 125.6, 128.3, 128.3, 143.1; HRMS (ESI) calcd. for $C_{15}H_{23}O$ $[M+H]^+$: 219.1749; found: 219.1752.

(2-(1-Ethyl-3-(methoxymethyl)cyclobutyl-2-d)ethyl)benzene (2c):

R_f = 0.50 (Hexane/Et_2O = 98:2); dr = >98:2:0:0; Yield: 80% (Colorless oil)

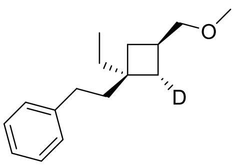

^{1}H NMR of major isomer (400 MHz, $CDCl_3$): 0.79 (t, *J* = 7.2 Hz, 3H), 1.41 (t, *J* = 9.2 Hz, 1H), 1.48-1.53 (m, 2H), 1.56-1.60 (m, 2H), 1.78-1.83 (m, 2H), 2.34-2.38 (m, 3H), 3.26-3.28(m, 5H), 7.08-7.11 (m, 3H), 7.19-7.22 (m, 2H); ^{13}C NMR of major isomer (100 MHz, $CDCl_3$): δ = 8.3, 28.1, 29.8, 30.2, 34.4 (t, *J* = 22 Hz, 1C), 34.9, 38.0, 42.0, 58.8, 78.6, 125.5, 128.3, 128.3, 143.2; HRMS (ESI) calcd. for $C_{16}H_{23}DO$ $[M+H]^+$: 234.1968; found: 234.1942.

(2-(1-Ethyl-2-iodo-3-(methoxymethyl)cyclobutyl)ethyl)benzene (2d):

R_f = 0.49 (Hexane/Et_2O = 97:3); dr = 92:8:0:0; Yield: 67% (Colorless oil)

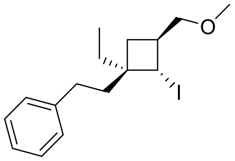

^{1}H NMR of major isomer (400 MHz, $CDCl_3$): δ = 0.92 (t, *J* = 7.6 Hz, 3H), 1.54-1.69 (m, 4H), 1.84 (dt, *J* = 5.2, 13.2 Hz, 1H), 1.94 (t, *J* = 10.0 Hz, 1H), 2.47 (dt, *J* = 4.4, 13.2 Hz, 1H), 2.63 (dt, *J* = 4.4, 13.2 Hz, 1H), 2.68-2.76 (m, 1H), 3.29-3.37 (m, 5H), 4.39 (d, *J* = 9.6 Hz, 1H), 7.14-7.17 (m, 3H), 7.24-7.27 (m, 2H); ^{13}C NMR of major isomer (100 MHz, $CDCl_3$): δ = 9.1, 29.4, 30.7 (x2), 33.3, 40.9, 43.4, 43.8, 59.3, 72.7, 125.9, 128.4, 128.6, 142.7; HRMS (ESI) calcd. for $C_{16}H_{24}OI$ $[M+H]^+$: 359.0872; found: 359.0870.

(2-(2-Bromo-1-ethyl-3-(methoxymethyl)cyclobutyl)ethyl)benzene (2e):

R_f = 0.45 (Hexane/Et_2O = 97:3); dr = 93:7:0:0; Yield: 58% (Colorless oil)

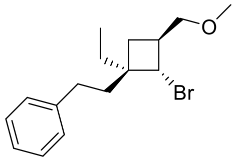

^{1}H NMR of major isomer (400 MHz, $CDCl_3$): δ = 0.87 (t, *J* = 7.6 Hz, 3H), 1.43 (t, *J* = 10.8 Hz, 1H), 1.54-1.59 (m, 2H), 1.71-1.81 (m, 2H), 1.96 (t, *J* = 10.0 Hz, 1H), 2.44 (dt, *J* = 4.8, 12.8 Hz, 1H), 2.55 (dt, *J* = 4.8, 12.8 Hz, 1H), 2.59-2.69 (m, 1H), 3.29-3.34 (m, 5H), 4.24 (d, *J* = 9.2 Hz, 1H), 7.09-7.11 (m, 3H), 7.19-7.22 (m, 2H); ^{13}C NMR of major isomer (100 MHz, $CDCl_3$): δ = 8.2, 26.6, 29.6, 31.8, 40.5, 42.6, 44.9, 54.6, 59.1, 72.7, 125.7, 128.2, 128.4, 142.5; HRMS (ESI) calcd. for $C_{16}H_{23}OBrNa$ $[M+Na]^+$: 333.0830; found: 333.0823.

(2-(2-Allyl-1-ethyl-3-(methoxymethyl)cyclobutyl)ethyl)benzene (2f):

R_f = 0.45 (Hexane/Et_2O = 98:2); dr = >98:2:0:0; Yield: 72% (Colorless oil)

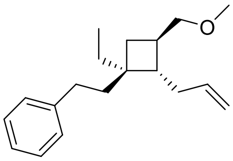

^{1}H NMR of major isomer (400 MHz, $CDCl_3$): δ = 0.78 (t, *J* = 7.2 Hz, 3H), 1.49-1.56 (m, 4H), 1.67-1.77 (m, 2H), 2.12-2.19 (m, 3H), 2.31-2.37 (m, *2*H), 2.54-2.56 (m, 1H), 3.23 (s, 3H), 3.25-3.29 (m, 1H), 3.42 (dd, *J* = 7.6, 9.2 Hz, 1H), 4.85 (d, *J* = 10.8 Hz, 1H), 4.92 (d, *J* = 17.2 Hz, 1H), 5.65-5.74 (m, 1H), 7.10-7.12 (m, 3H), 7.19-7.23 (m, 2H); ^{13}C NMR of major isomer (100 MHz, $CDCl_3$): δ = 8.3, 29.7, 30.4, 31.3, 31.3, 33.9, 35.4, 41.1, 43.2, 58.6, 74.5, 114.7, 125.6, 128.3, 128.4, 138.9, 143.4; HRMS (ESI) calcd. for $C_{19}H_{28}ONa$ $[M+Na]^+$: 295.2038; found: 295.2027.

(2-Allyl-3-ethyl-3-phenethylcyclobutyl)methanol (2g):

R_f = 0.48 (Hexane/EtOAc = 8:2); dr = 9:1:0:0; Yield: 66% (Colorless oil)

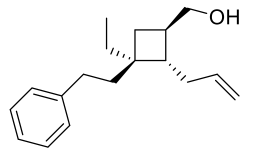

^{1}H NMR of major isomer (400 MHz, $CDCl_3$): δ = 0.80 (t, *J* = 7.2 Hz, 3H), 1.31-1.33 (m, 1H), 1.34-1.44 (m, 1H), 1.47-1.60 (m, 2H), 1.63-1.75 (m, 2H), 2.13-2.20 (m, 3H), 2.23-2.36 (m, 2H), 2.37-2.53 (m, 1H), 3.44-3.48 (m, 1H), 3.68-3.72 (m, 1H), 4.94 (d, *J* = 10.4 Hz, 1H), 5.02 (d, *J* = 16.4 Hz, 1H), 5.74-5.76 (m, 1H), 7.09-7.12 (m, 3H), 7.18-7.23 (m, 2H); ^{13}C NMR of major isomer (100 MHz, $CDCl_3$): δ = 8.3, 29.7, 30.4, 31.3, 33.1, 34.0, 35.5, 40.5, 42.7, 64.3, 115.2, 125.7, 128.2, 128.4, 139.4, 143.2; HRMS (ESI) calcd. for $C_{18}H_{27}O$ $[M+H]^+$: 259.2056; found: 259.2043.

Ethyl 2-((2-ethyl-4-(methoxymethyl)-2-phenethylcyclobutyl)methyl)acrylate (2h):

R_f = 0.44 (Hexane/Et_2O = 9:1); dr = >98:2:0:0; Yield: 71% (Colorless oil)

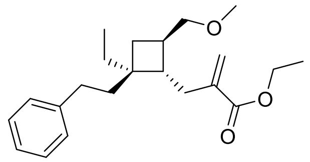

^{1}H NMR of major isomer (400 MHz, $CDCl_3$): δ = 0.80 (t, *J* = 7.2 Hz, 3H), 1.21 (t, *J* = 6.8 Hz, 3H), 1.49-1.64 (m, 5H), 1.69-1.78 (m, 1H), 2.31-2.45 (m, 5H), 2.54-2.60 (m, 1H), 3.21 (s, 3H), 3.27-3.31 (m, 1H), 3.37-3.41 (m, 1H), 4.09-4.15 (m, 2H), 5.45 (d, *J* = 0.8 Hz, 1H), 6.09 (s, 1H), 7.09-7.13 (m, 3H), 7.19-7.24 (m, 2H); ^{13}C NMR of major isomer (100 MHz, $CDCl_3$): δ = 8.5, 14.4, 27.7, 29.9, 31.6, 32.0, 34.2, 35.6, 41.5, 41.7, 58.9, 60.8, 74.6, 124.5, 125.8, 128.5, 128.6, 140.8, 143.5, 167.7; HRMS (ESI) calcd. for $C_{22}H_{33}O_3$ $[M+H]^+$: 345.2430; found: 345.2423.

1-(2-Ethyl-4-(methoxymethyl)-2-phenethylcyclobutyl)-3-methoxybenzene (2i):

R_f = 0.45 (Hexane/Et_2O = 9:1); dr = 93:7:0:0; Yield: 58% (Colorless oil)

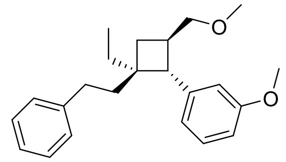

^{1}H NMR of major isomer (400 MHz, $CDCl_3$): δ = 0.92 (t, *J* = 7.6 Hz, 3H), 1.56-1.61 (m, 2H), 1.70-1.82 (m, 3H), 1.89-1.97 (m, 1H), 2.01-2.06 (m, 1H), 2.17-2.24 (m, 1H), 2.93-2.99 (m, 1H), 3.14 (s, 3H), 3.25 (dd, *J* = 2, 9.2 Hz, 1H), 3.35 (dd, *J* = 6.8, 9.8 Hz, 1H), 3.46 (dd, *J* = 7.6, 9.6 Hz, 1H), 3.81 (s, 3H), 6.72-6.80 (m, 3H), 6.82 (d, *J* = 7.6 Hz, 1H), 6.88-6.90 (m, 1H), 7.04-7.09 (m, 1H), 7.11-7.14 (m, 2H), 7.19-7.23 (m, 1H); ^{13}C NMR of major isomer (100 MHz, $CDCl_3$): δ = 8.8, 30.2, 31.4, 33.6, 35.8, 36.0, 43.4, 53.1, 55.4, 58.9, 74.2, 111.1, 117.0, 123.2, 125.6, 128.3, 128.4, 128.7, 141.4, 143.4, 159.3; HRMS (ESI) calcd. for $C_{23}H_{31}O_2$ $[M+H]^+$: 339.2324; found: 339.2360.

2-Ethyl-4-(methoxymethyl)-2-phenethylcyclobutan-1-ol (2j):

R_f = 0.51 (Hexane/EtOAc = 8:2); dr = >98:2:0:0; Yield: 67% (Colorless oil)

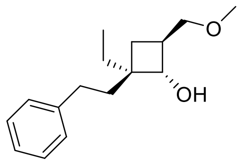

^{1}H NMR of major isomer (400 MHz, $CDCl_3$): δ = 0.85-0.95 (m, 4H), 1.44-1.55 (m, 3H), 1.63-1.68 (m, 2H), 1.73-1.78 (m, 1H), 2.21-2.28 (m, 1H), 2.47-2.51 (m, 2H), 3.27 (s, 3H), 3.33 (d, *J* = 6.4 Hz, 2H), 3.66 (d, *J* = 8.0 Hz, 1H), 7.09-7.14 (m, 3H), 7.19-7.23 (m, 2H); ^{13}C NMR of major isomer (100 MHz, $CDCl_3$): δ = 7.8, 22.2, 27.6, 30.6, 40.2, 40.8, 45.1, 58.9, 75.3, 76.9, 125.8, 128.3, 128.4, 142.8; HRMS (ESI) calcd. for $C_{16}H_{24}O_2Na$ $[M+Na]^+$: 271.1674; found: 271.1682.

1-Ethyl-1-hexyl-3-(methoxymethyl)cyclobutane-2-d (2k):

R_f = 0.55 (Hexane/Et_2O = 98:2); dr = >98:2:0:0; Yield: 80% (Colorless oil)

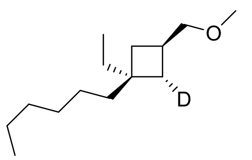

^1H NMR of major isomer (400 MHz, $CDCl_3$): δ = 0.76 (t, *J* = 7.2 Hz, 3H), 0.85 (t, *J* = 6.4 Hz, 3H), 1.05-1.11 (m, 2H), 1.23-1.29 (m, 8H), 1.35-1.55 (m, 3H), 1.76-1.81 (m, 2H), 2.36-2.42 (m, 1H), 3.30-3.37 (m, 5H); ^{13}C NMR of major isomer (100 MHz, $CDCl_3$): δ = 8.5, 14.3, 22.9, 23.8, 28.3, 30.1, 30.2, 32.2, 34.78 (t, *J* = 19.7 Hz, 1C), 35.2, 38.2, 40.1, 59.0, 79.0; HRMS (ESI) calcd. for $C_{14}H_{27}DONa$ $[M+Na]^+$: 236.2101; found: 236.2106.

(3-Ethyl-3-hexylcyclobutyl)methanol (2l):

R_f = 0.5 (Hexane/EtOAc = 8:2); dr = 94:6:0:0; Yield: 69% (Colorless oil)

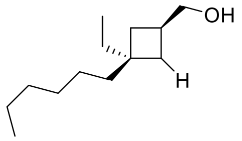

^1H NMR of major isomer (400 MHz, $CDCl_3$): 0.76 (t, *J* = 7.2 Hz, 3H), 0.85 (t, *J* = 6.8 Hz, 3H), 1.05-1.11 (m, 2H), 1.19-1.29 (m, 8H), 1.35-1.47 (m, 5H), 1.73-1.78 (m, 2H), 1.30-1.38 (m, 1H), 3.54 (d, *J* = 6.8 Hz, 2H); ^{13}C NMR of major isomer (100 MHz, $CDCl_3$): δ = 8.4, 14.3, 22.9, 23.8, 30.2, 30.2, 30.7, 32.2, 34.4 (x2), 38.0, 40.0, 68.6; HRMS (ESI) calcd. for $C_{13}H_{27}O$ $[M+H]^+$: 199.2062; found: 199.2068.

(2-allyl-3-ethyl-3-hexylcyclobutyl)methanol (**2m**):

R_f = 0.39 (Hexane/Et_2O = 90:10); d.r. = 9:1:0:0; Yield = 50% (Pale yellow oil)

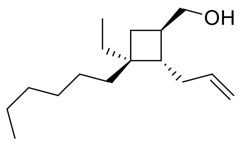

^1H NMR of major isomer (400 MHz, $CDCl_3$) δ 0.77 (t, *J* = 7.4 Hz, 3H), 0.88 (t, *J* = 6.8 Hz, 3H), 1.07 (m, 2H), 1.21 – 1.31 (m, 7H), 1.33 – 1.56 (m, 5H), 1.70 (t, *J* = 10.2 Hz, 1H), 2.14 – 2.37 (m, 3H), 2.54 (dq, *J* = 8.5, 16.9 Hz, 1H), 3.49 (dd, *J* = 6.3, 11.1 Hz, 1H), 3.77 (dd, *J* = 8.7, 11.1 Hz, 1H), 5.01 (d, *J* = 10.2 Hz, 1H), 5.10 (d, *J* = 17.2 Hz, 1H), 5.84 (m, 1H); ^{13}C NMR of major isomer (100 MHz, $CDCl_3$) δ 8.3, 14.1, 22.7, 23.1, 30.2, 30.4, 31.4, 32.0, 33.0, 33.1, 34.0, 40.4, 42.6, 64.3, 115.0, 139.7; HRMS (ESI) calcd. for $C_{16}H_{31}O$ $[M+H]^+$: 239.2375; found 239.2368.

1-(((3-Ethyl-3-hexylcyclobutyl-2-d)methoxy)methyl)-4-methoxybenzene (2n):

R_f = 0.53 (Hexane/Et_2O = 98:2); dr = >98:2:0:0; Yield: 69% (Colorless oil)

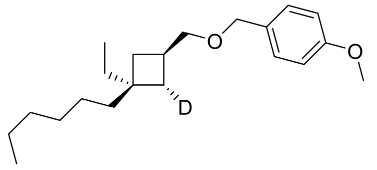

^1H NMR of major isomer (400 MHz, $CDCl_3$): δ = 0.74 (t, *J* = 7.2 Hz, 3H), 0.85 (t, *J* = 6.8 Hz, 3H), 1.03-1.09 (m, 2H), 1.20-1.27 (m, 8H), 1.34-1.45 (m, 3H), 1.74-1.79 (m, 2H), 2.40-2.42 (m, 1H), 3.35 (d, *J* = 7.2 Hz, 2H), 3.77 (s, 3H), 4.40 (s, 2H), 6.84 (d, *J* = 8.8 Hz, 2H), 7.22 (d, *J* = 8.8 Hz, 2H); ^{13}C NMR of major isomer (100 MHz, $CDCl_3$): δ = 8.5, 14.3, 22.9, 23.8, 28.4, 30.2, 30.2, 32.2, 34.8 (d, *J* = 19.7 Hz, 1C), 35.2, 38.1, 40.0, 55.5, 72.7, 76.0, 113.9, 129.4, 131.1, 159.3; HRMS (ESI) calcd. for $C_{21}H_{33}DO_2Na$ $[M+Na]^+$: 342.2519; found: 342.2516.

Ethyl 2-((2-ethyl-2-hexyl-4-(methoxymethyl)cyclobutyl)methyl)acrylate (2o):

R_f = 0.46 (Hexane/Et_2O = 9:1); dr = 92:8:0:0; Yield: 69% (Colorless oil)

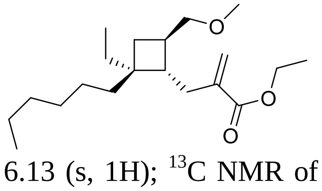

^1H NMR of major isomer (400 MHz, $CDCl_3$): δ = 0.73 (t, *J* = 7.2 Hz, 3H), 0.83-0.87 (m, 3H), 1.05-1.07 (m, 2H), 1.19-1.33 (m, 9H), 1.37-1.52 (m, 5H), 1.69-1.74 (m, 1H), 2.36-2.47 (m, 3H), 2.53-2.60 (m, 1H), 3.25 (s, 3H), 3.29-3.33 (m, 1H), 3.40-3.44 (m, 1H), 4.16-4.21 (m, 2H), 5.50 (d, *J* = 0.8 Hz, 1H), 6.13 (s, 1H); ^{13}C NMR of major isomer (100 MHz, $CDCl_3$): δ = 8.5, 14.3, 14.4, 22.9, 23.2, 27.6, 30.4, 31.5, 32.0, 32.1, 33.1, 34.3, 41.4, 41.5, 58.9, 60.8, 74.7, 124.4, 140.9, 167.8; HRMS (ESI) calcd. for $C_{20}H_{37}O_3$ $[M+H]^+$: 325.2743; found: 325.2738.

2-Ethyl-2-hexyl-4-(((4-methoxybenzyl)oxy)methyl)cyclobutan-1-ol (2p):

R_f = 0.50 (Hexane/EtOAc = 9:1); dr = 9:1:0:0; Yield: 51% (Colorless oil)

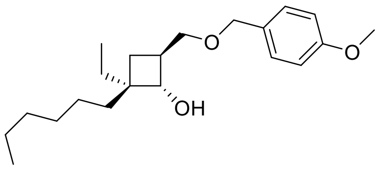

^{1}H NMR of major isomer (400 MHz, $CDCl_3$): δ = 0.77-0.82 (m, 6H), 0.89 (t, *J* = 10.0 Hz, 1H), 1.14-1.18 (m, 9H), 1.35-1.40 (m, 2H), 1.55-1.64 (m, 3H), 2.21-2.27 (m, 1H), 3.38-3.40 (m, 2H), 3.65 (t, *J* = 7.2 Hz, 1H), 3.73 (s, 3H), 4.38 (s, 2H), 6.80 (d, *J* = 8.8 Hz, 2H), 7.19 (d, *J* = 8.8 Hz, 2H); ^{13}C NMR of major isomer (100 MHz, $CDCl_3$): δ = 7.8, 14.1, 22.5, 22.7, 23.9, 27.8, 30.0, 31.9, 38.3, 41.2, 45.0, 55.3, 72.6, 72.7, 77.2, 113.8, 129.2, 130.7, 159.1; HRMS (ESI) calcd. for $C_{21}H_{34}O_3Na$ $[M+Na]^+$: 357.2406; found: 357.2402.

1-(2-Ethyl-2-hexyl-4-(methoxymethyl)cyclobutyl)-4-methoxybenzene (2q):

R_f = 0.47 (Hexane/Et_2O = 9:1); dr = 92:8:0:0; Yield: 61% (Colorless oil)

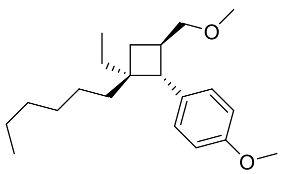

^{1}H NMR of major isomer (400 MHz, $CDCl_3$): δ = 0.75-0.83 (m, 6H), 0.90-1.02 (m, 4H), 1.09-1.16 (m, 3H), 1.23-1.30 (m, 3H), 1.63-1.71 (m, 3H), 1.92-1.97 (m, 1H), 2.86-2.92 (m, 1H), 3.12 (s, 3H), 3.17 (dd, *J* = 2.0, 9.6 Hz, 1H), 3.30 (dd, *J* = 6.4, 9.6 Hz, 1H), 3.41 (dd, *J* = 7.6, 9.6 Hz, 1H), 3.78 (s, 3H), 6.79 (d, *J* = 8.8 Hz, 2H), 7.14 (d, *J* = 8.8 Hz, 2H); ^{13}C NMR of major isomer (100 MHz, $CDCl_3$): δ = 8.8, 14.3, 22.7, 23.1, 30.0, 31.5, 31.8, 32.9, 33.7, 36.3, 43.3, 51.8, 55.4, 58.9, 74.4, 113.1, 131.5, 132.1, 157.9; HRMS (ESI) calcd. for $C_{21}H_{35}O_2$ $[M+H]^+$: 319.2637; found: 319.2650.

^{1}H NMR spectrum of 1a (400 MHz, $CDCl_3$):

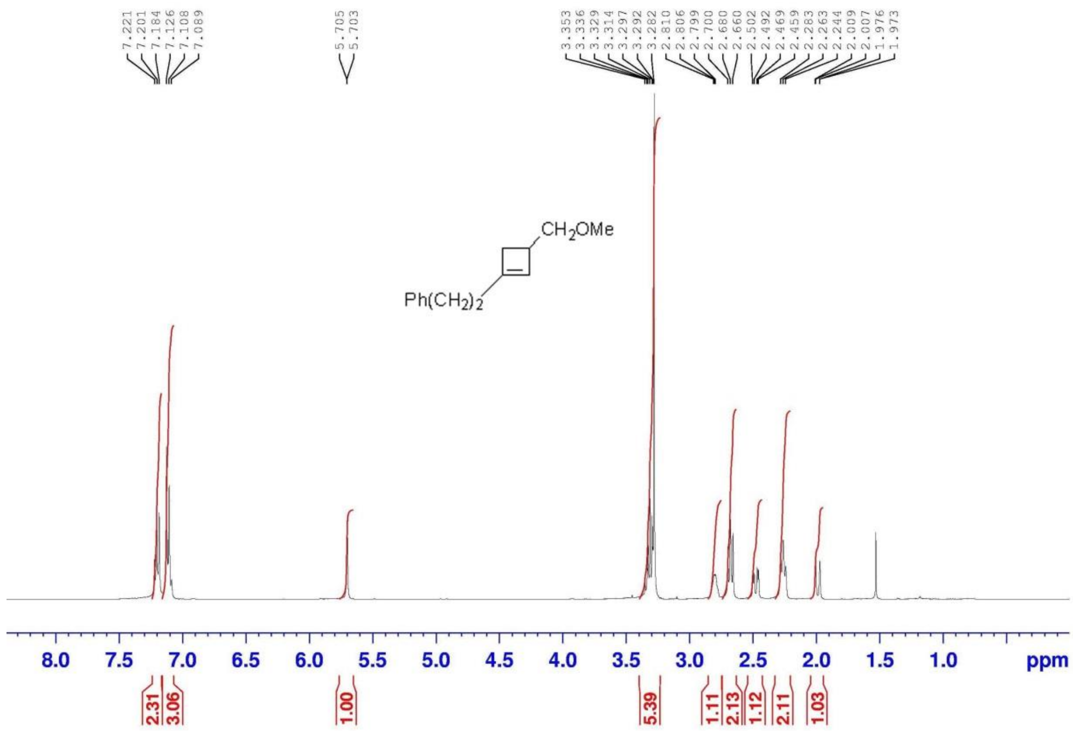

^{13}C NMR spectrum of 1a (100 MHz, $CDCl_3$):

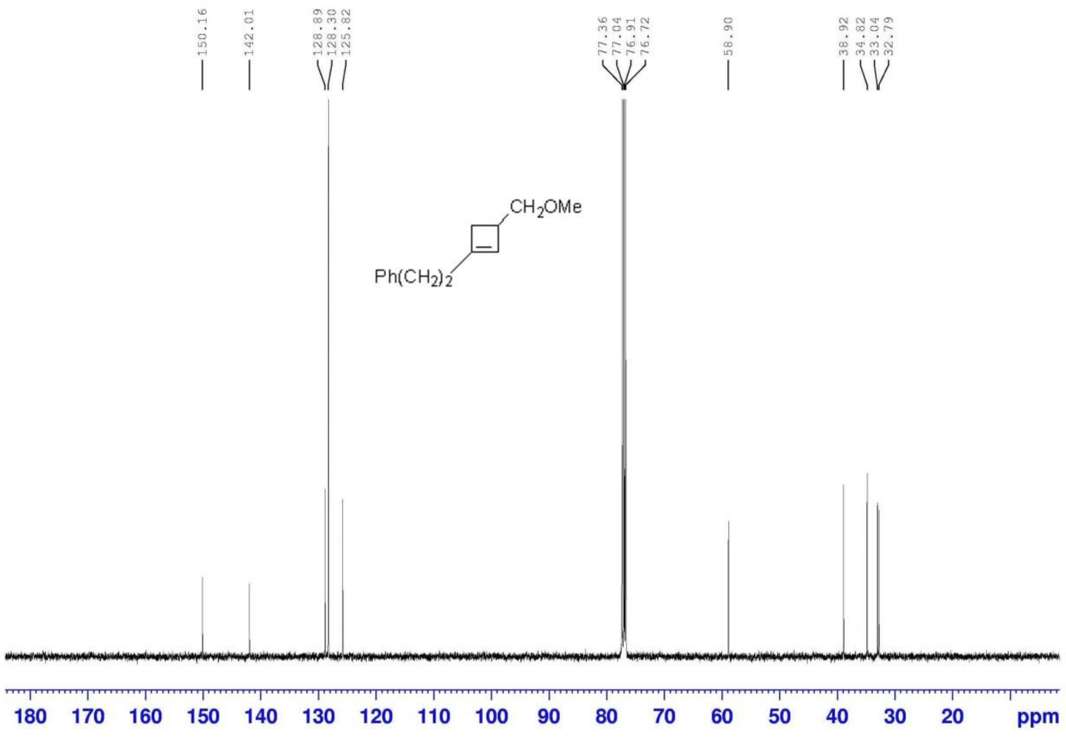

^{1}H NMR spectrum of 1b (400 MHz, $CDCl_3$):

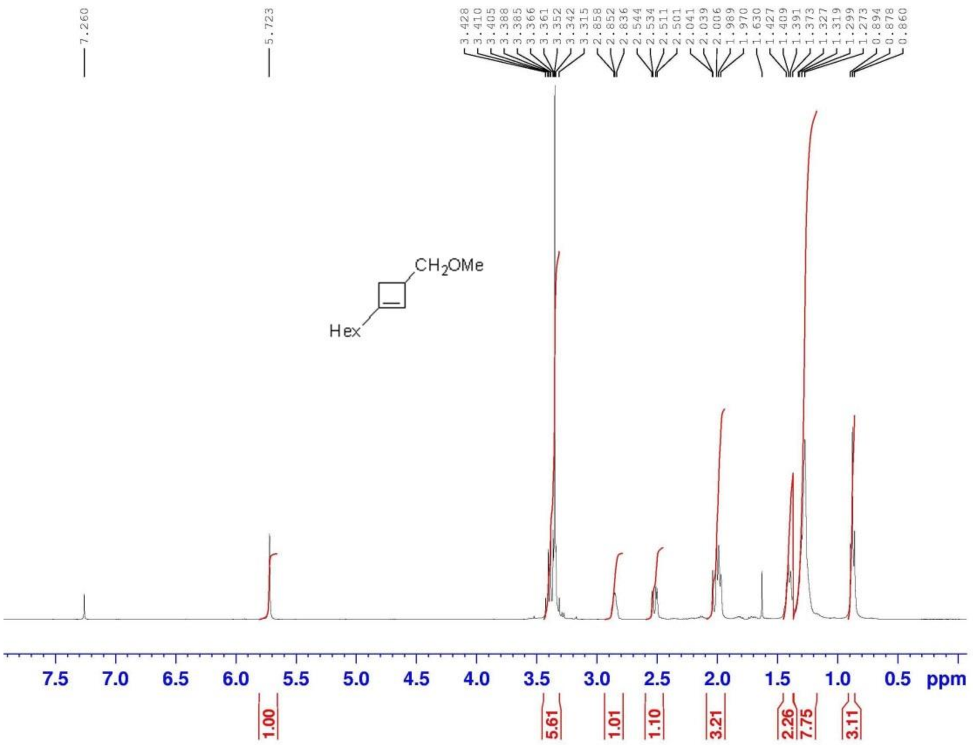

^{13}C NMR spectrum of 1b (100 MHz, $CDCl_3$):

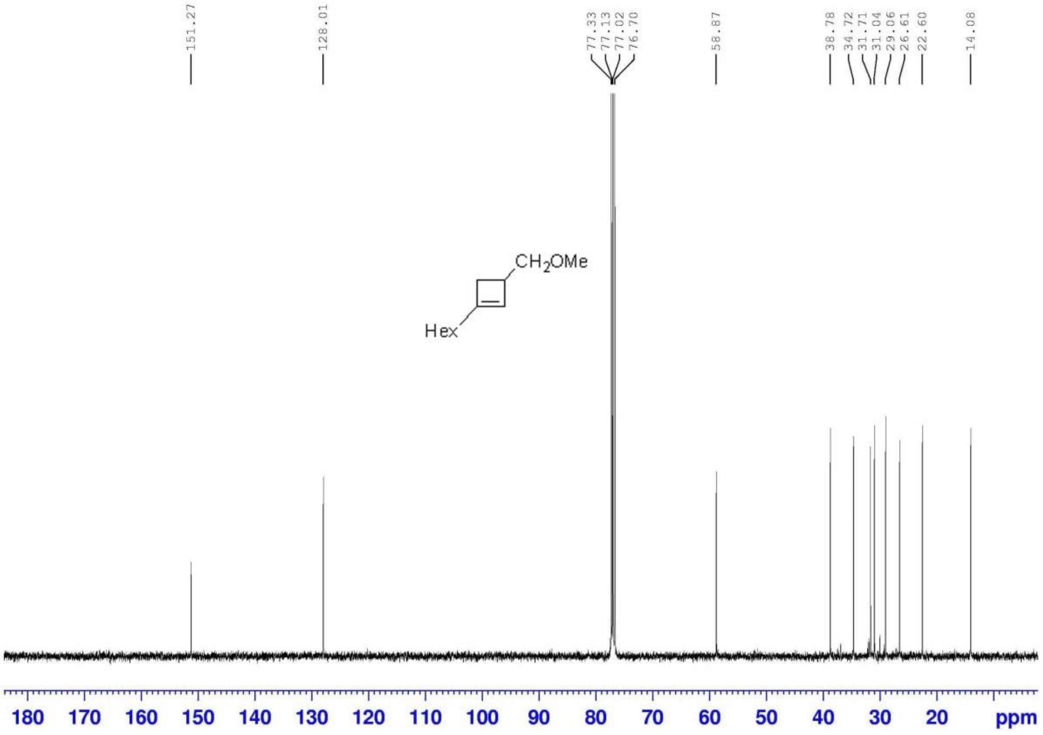

^{1}H NMR spectrum of 1e (400 MHz, $CDCl_3$):

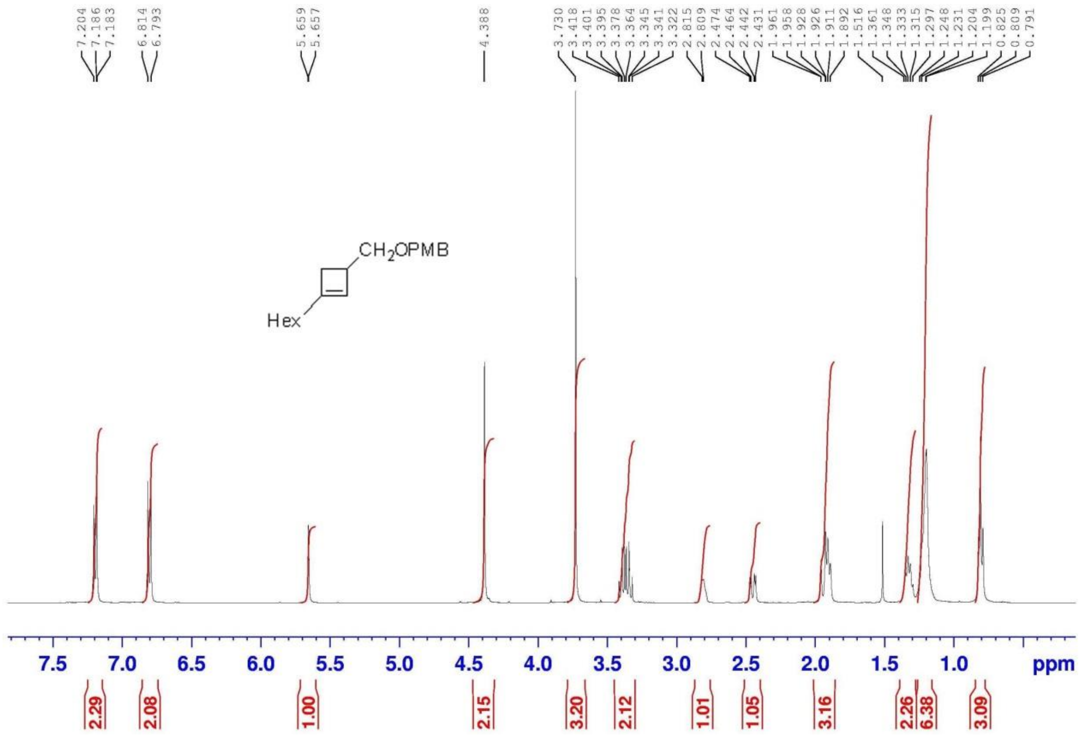

^{13}C NMR spectrum of 1e (100 MHz, $CDCl_3$):

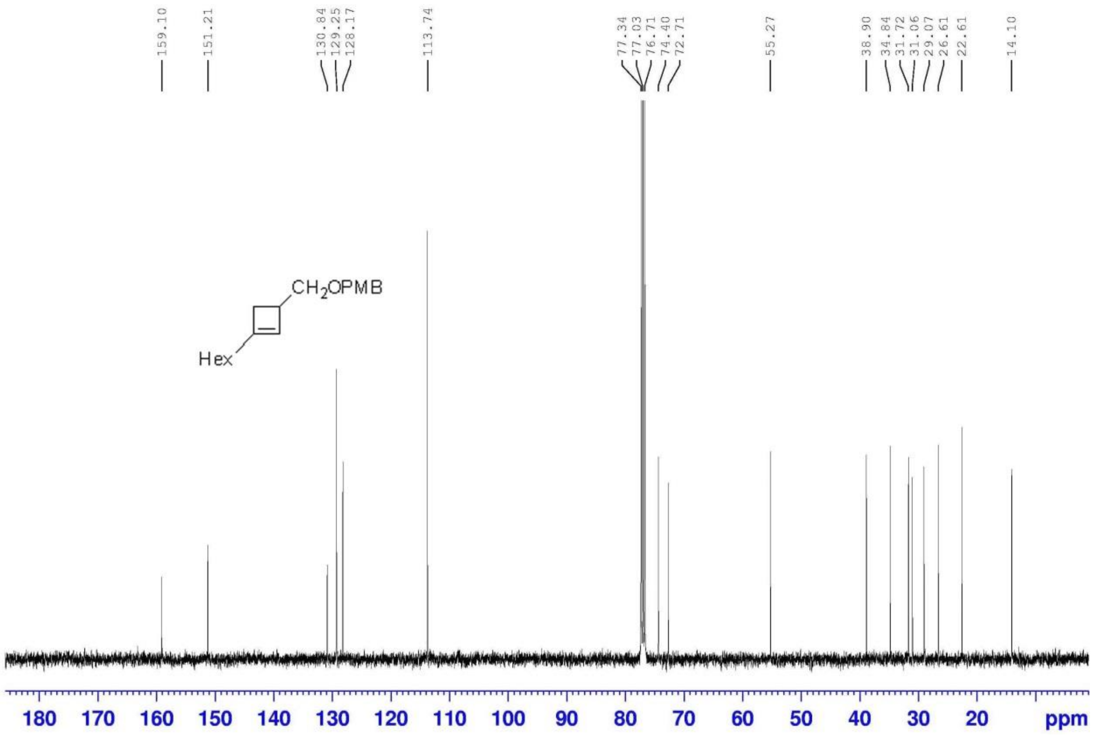

^{1}H NMR spectrum of 2a (400 MHz, $CDCl_3$):

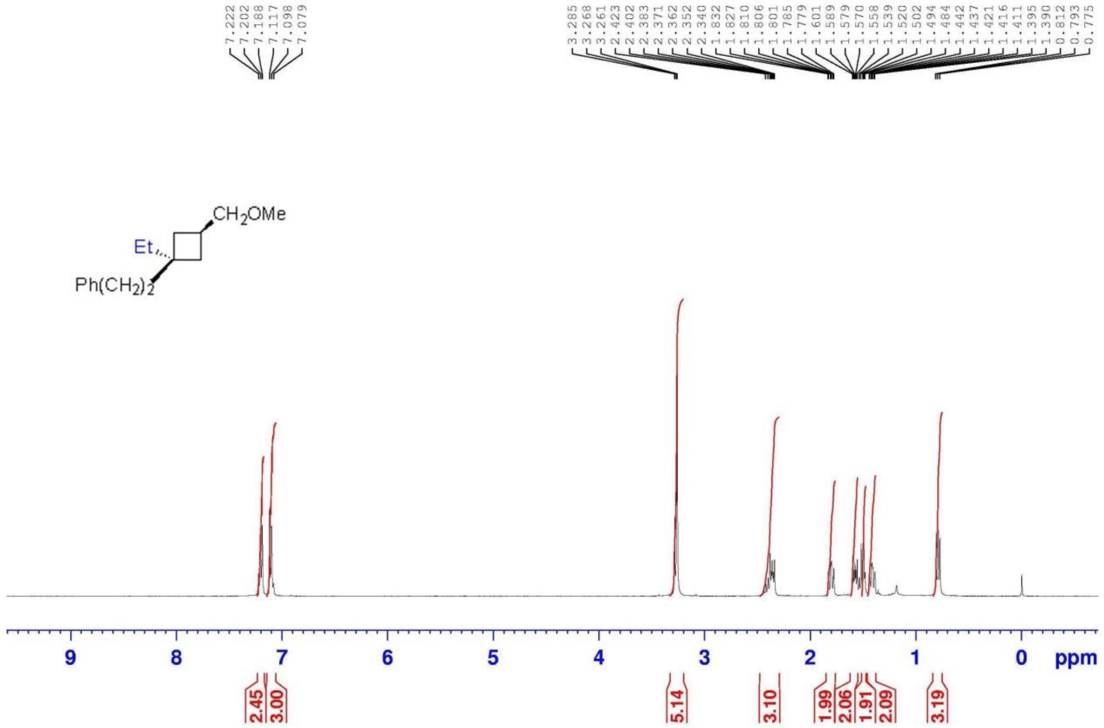

^{13}C NMR spectrum of 2a (100 MHz, $CDCl_3$):

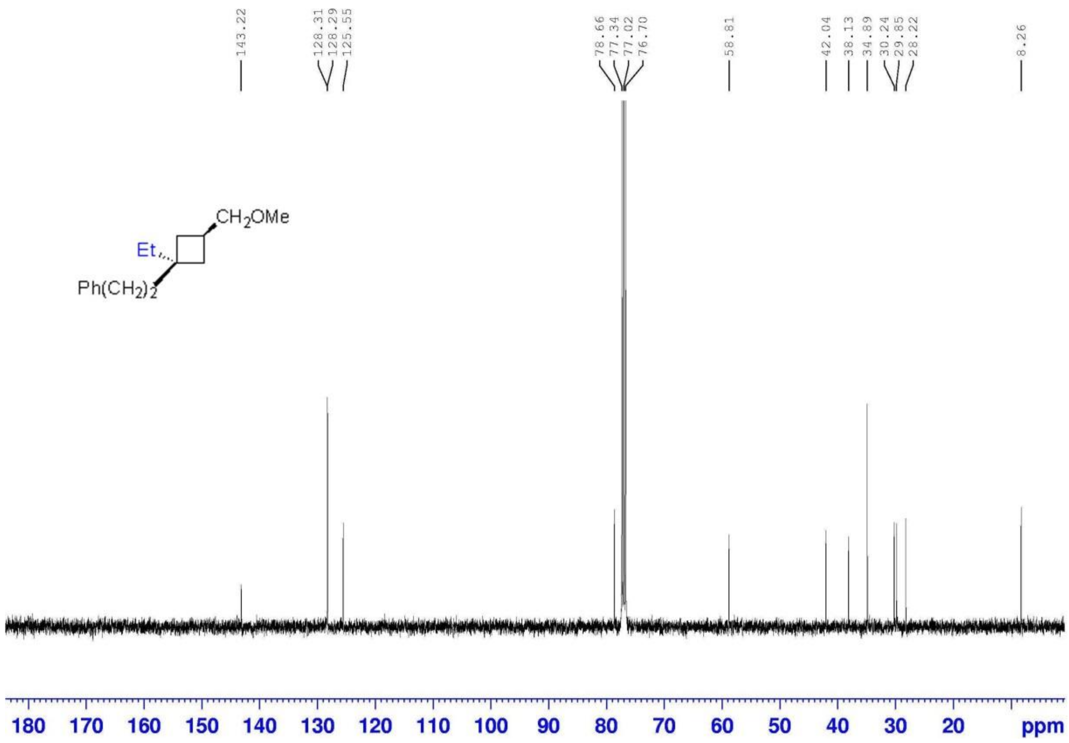

^{1}H-^{1}H NOE spectrum of 2a (600 MHz, $CDCl_3$):

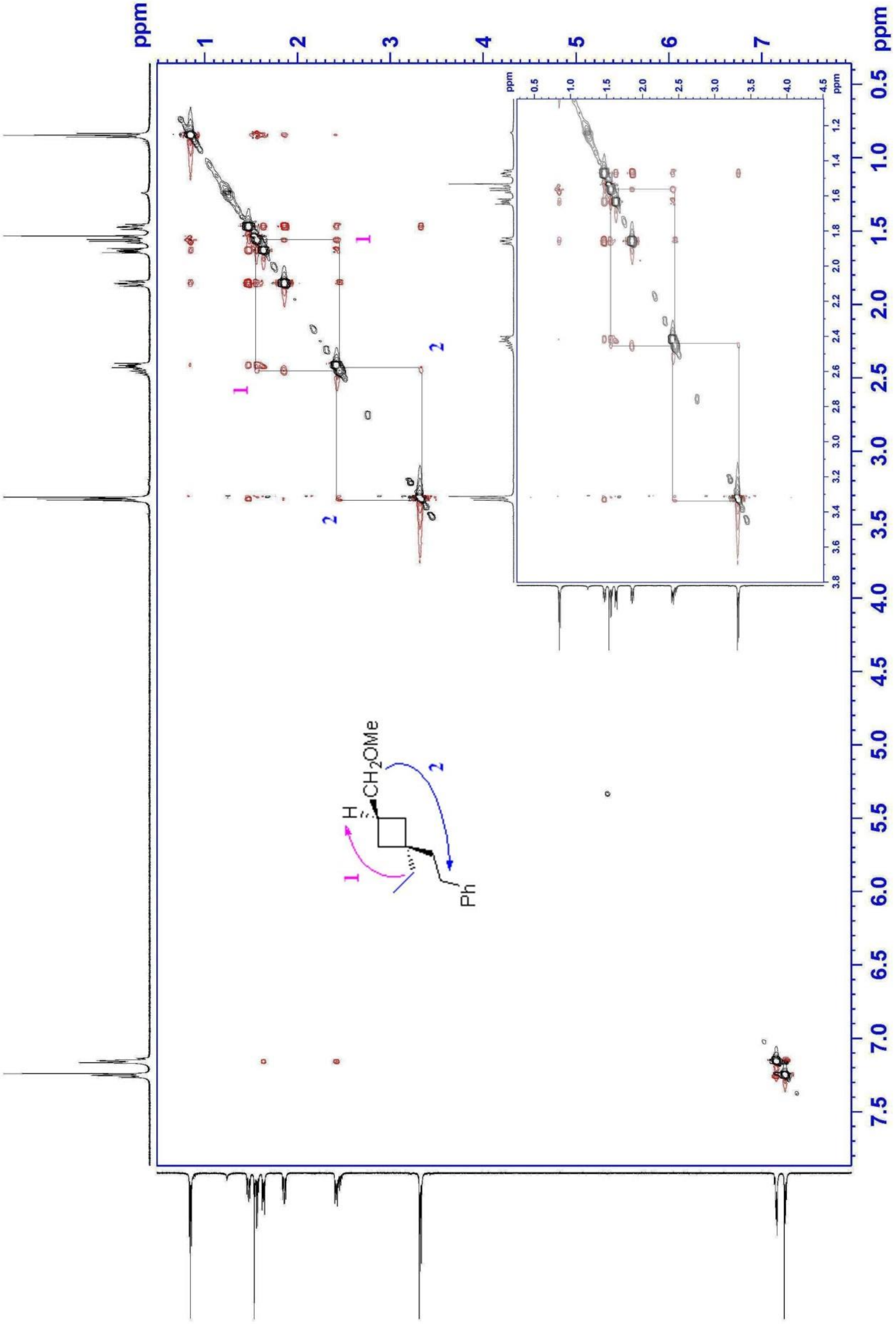

^{1}H NMR spectrum of 3a (400 MHz, $CDCl_3$):

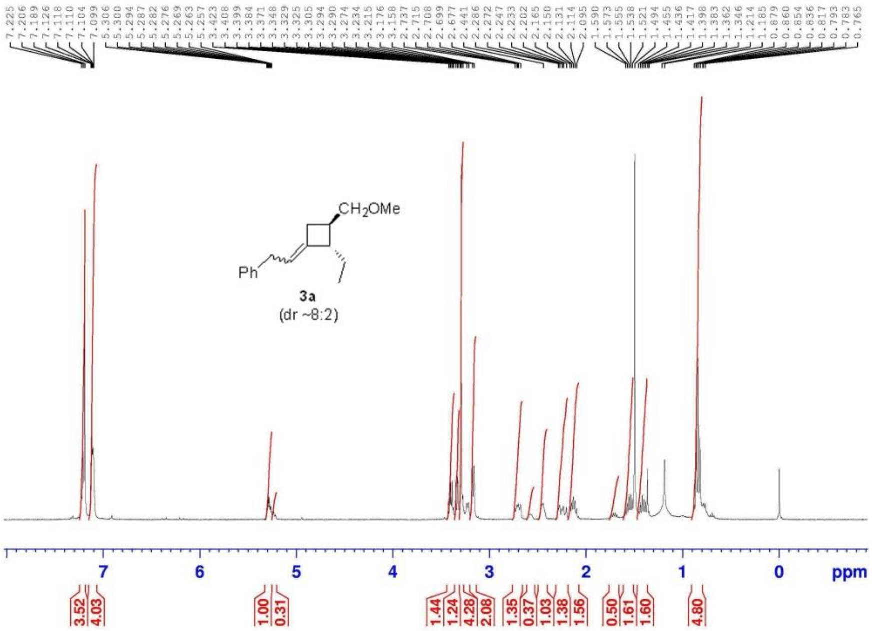

^{13}C NMR spectrum of 3a (100 MHz, $CDCl_3$):

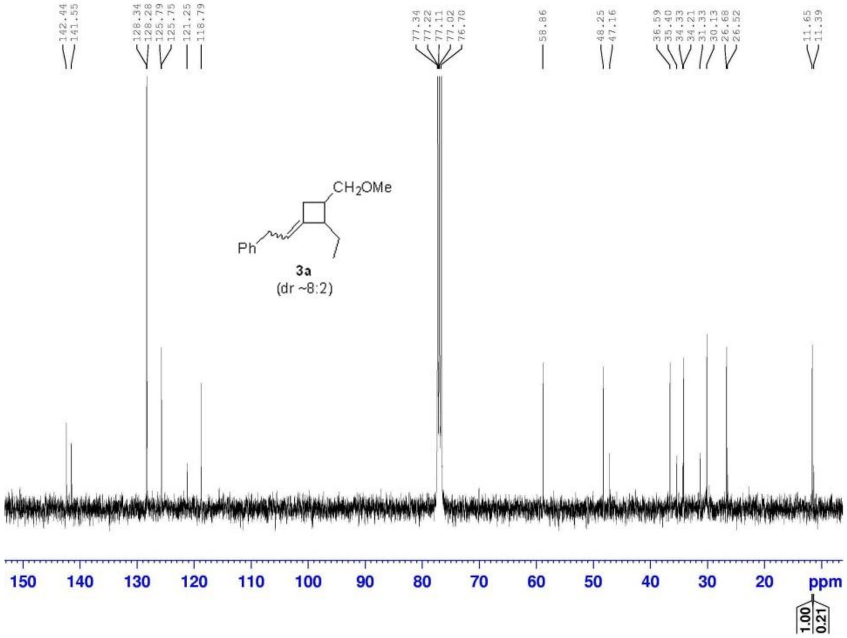

HRMS of 3a:

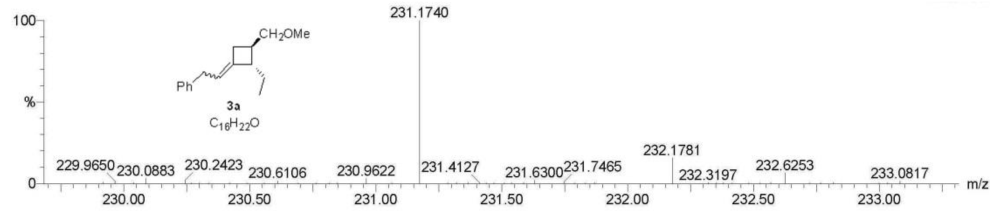

Minimum:				-1.5		
Maximum:		20.0	10.0	50.0		
Mass	Calc. Mass	mDa	PPM	DBE	i-FIT	Formula
231.1740	231.1749	-0.9	-3.9	5.5	51.0	C16 1H23 O

^{1}H NMR spectrum of 2b (400 MHz, $CDCl_3$):

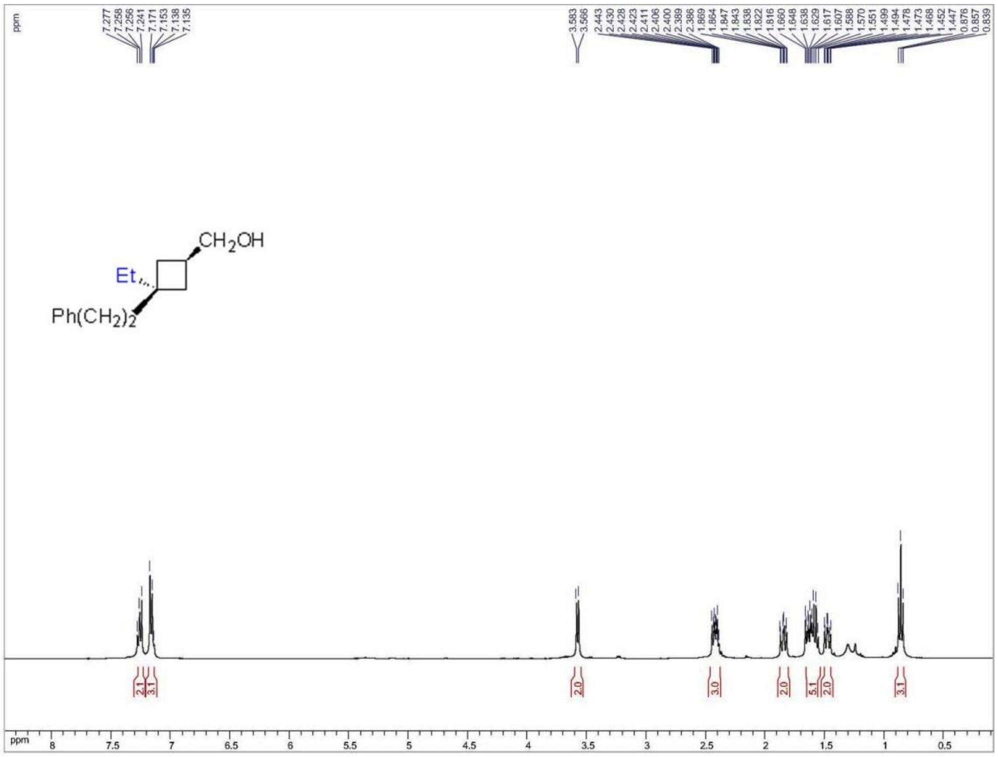

^{13}C NMR spectrum of 2b (100 MHz, $CDCl_3$):

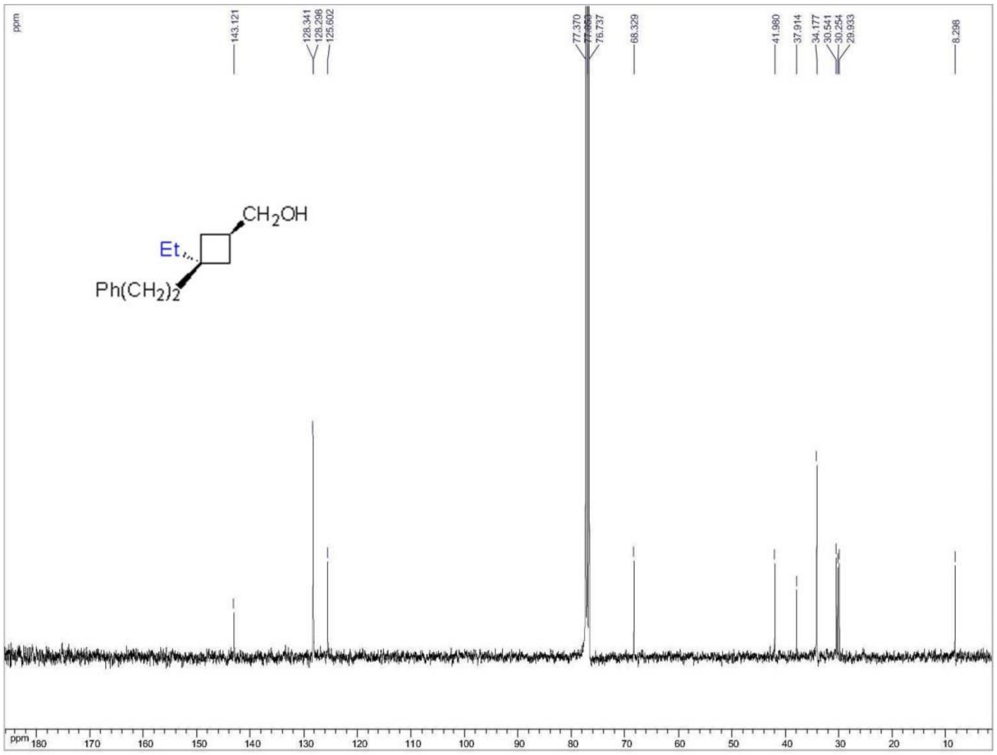

^{1}H NMR spectrum of 2c (400 MHz, $CDCl_3$):

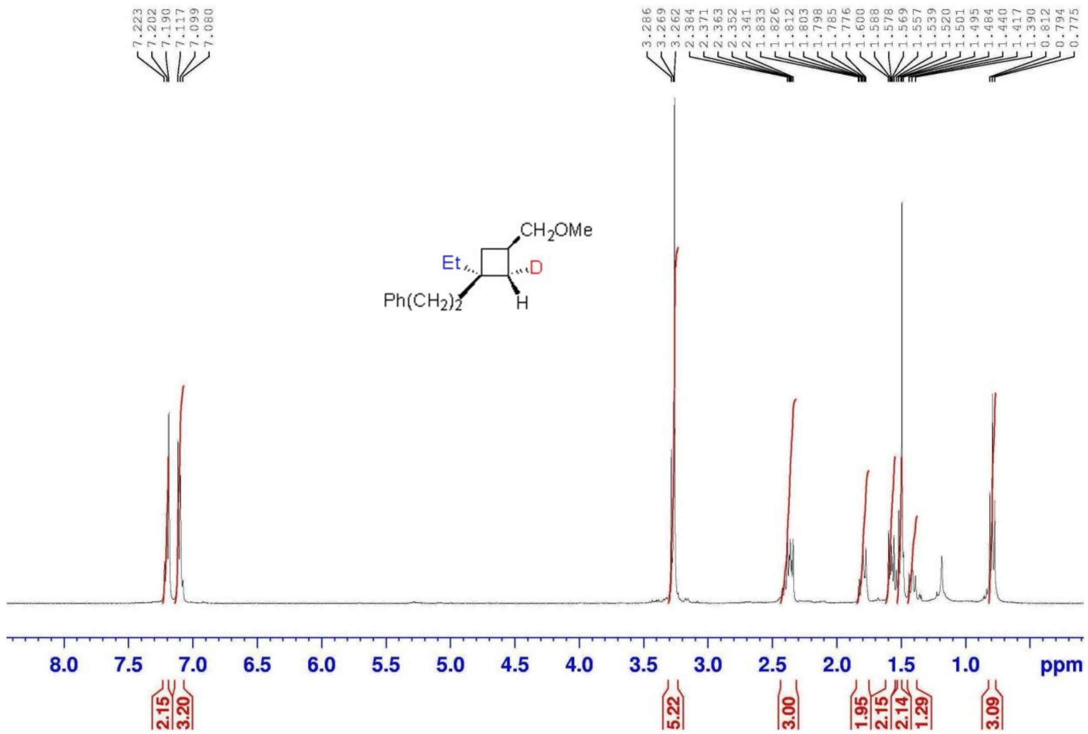

^{13}C NMR spectrum of 2c (100 MHz, $CDCl_3$):

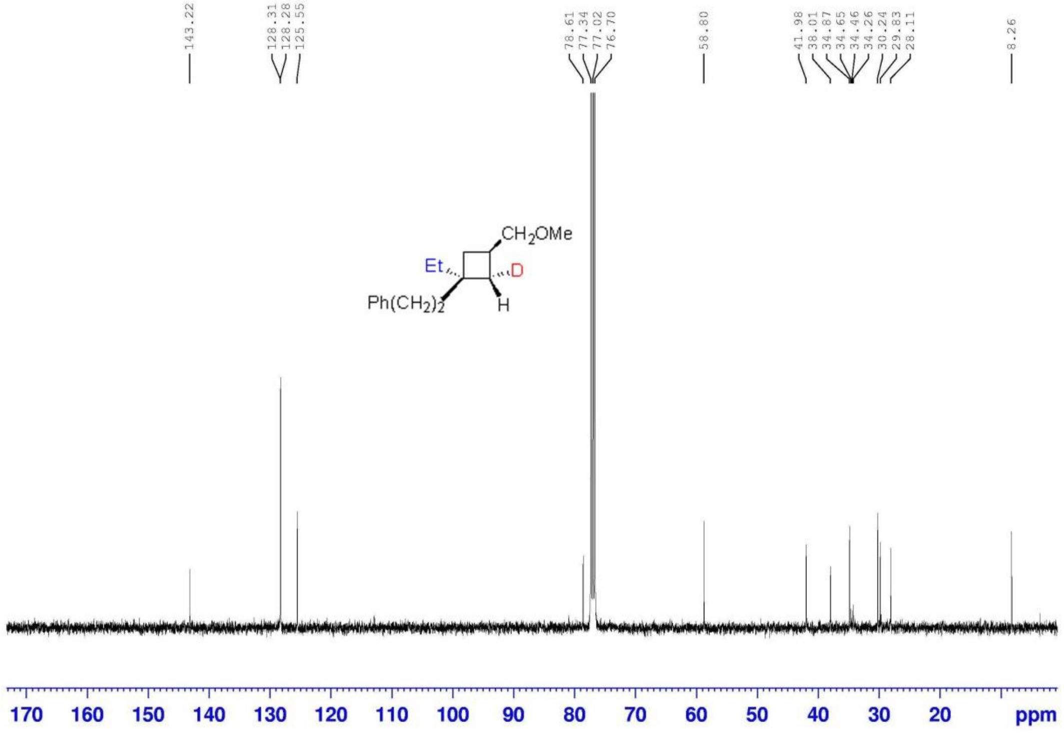

^{1}H NMR spectrum of 2d (400 MHz, $CDCl_3$):

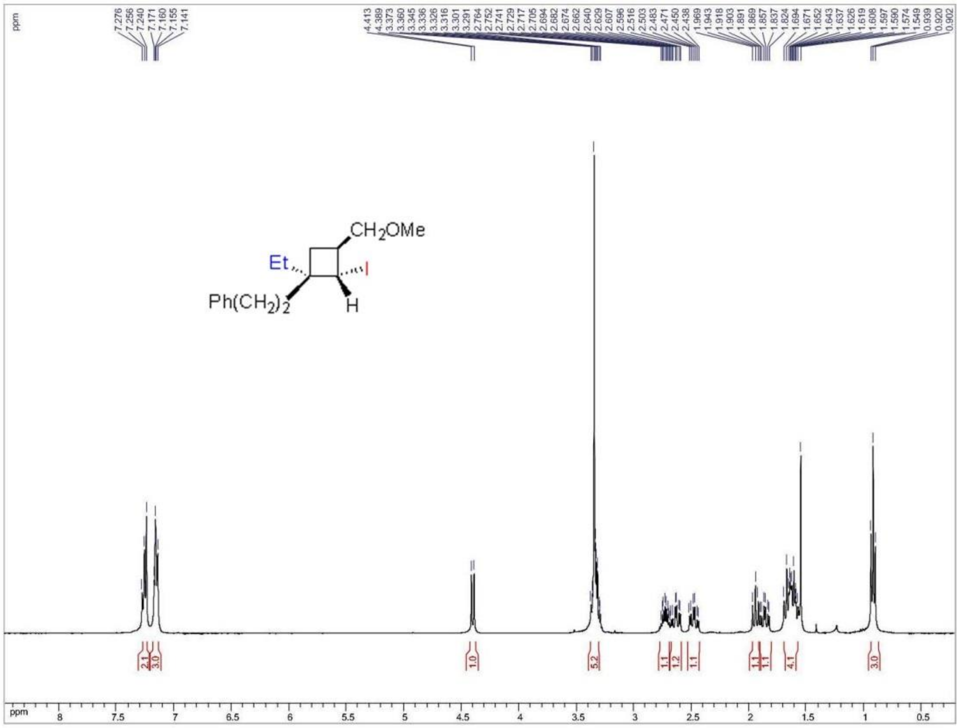

^{13}C NMR spectrum of 2d (100 MHz, $CDCl_3$):

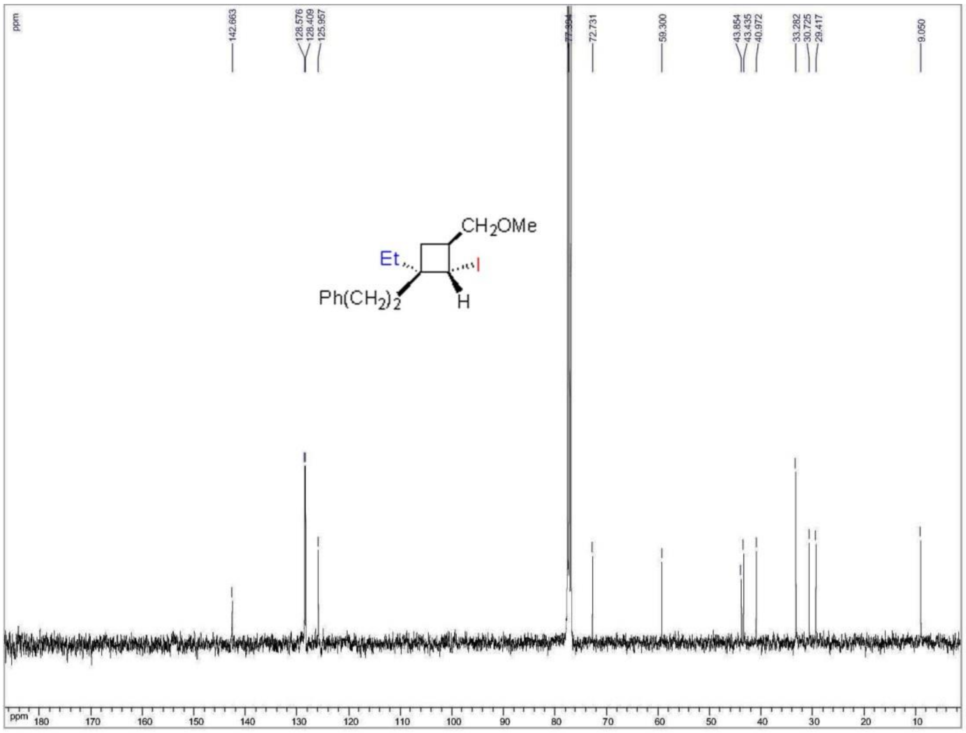

^{1}H-^{1}H NOE spectrum of 2d (600 MHz, $CDCl_3$):

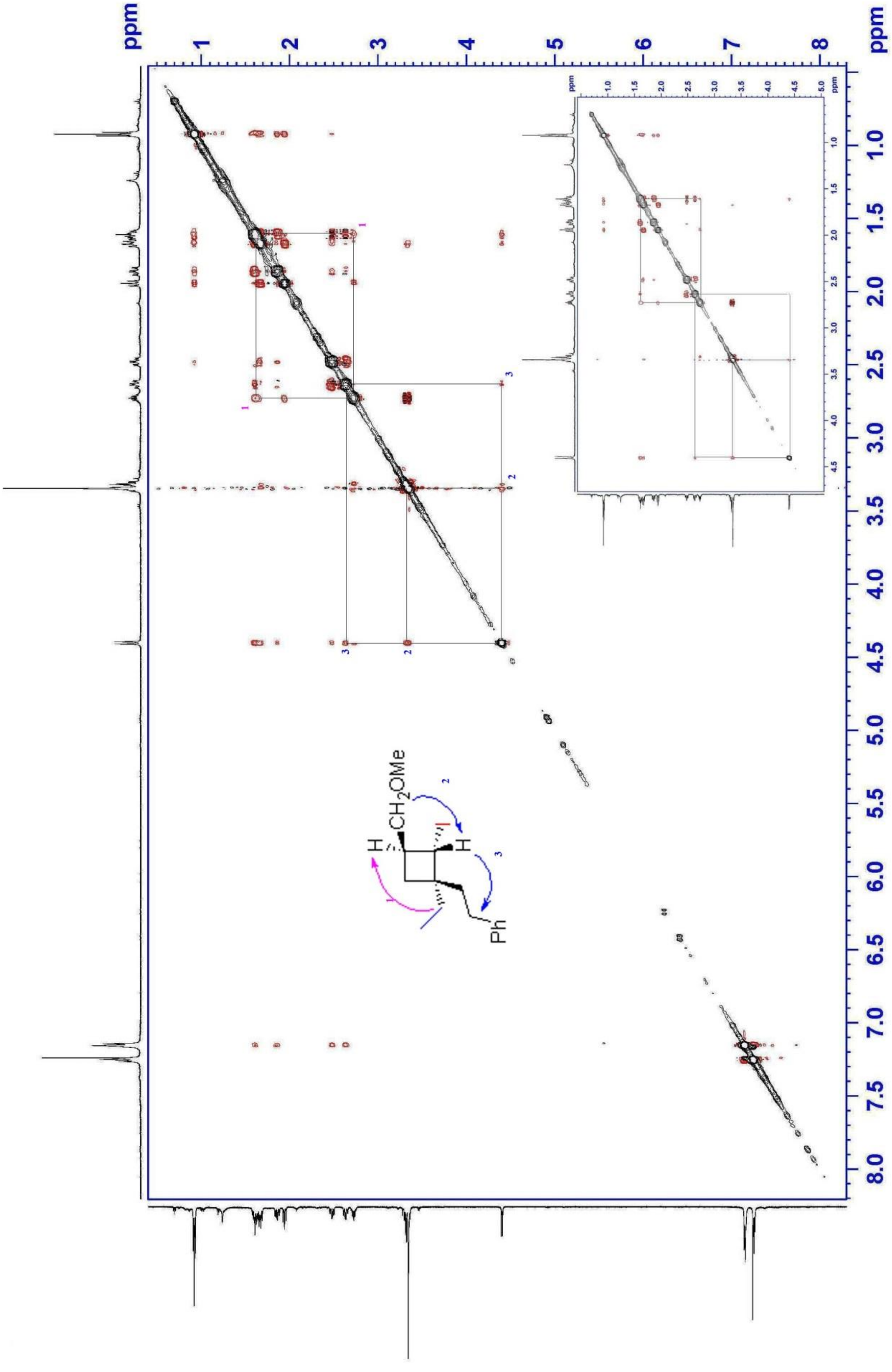

^{1}H NMR spectrum of 2e (400 MHz, $CDCl_3$):

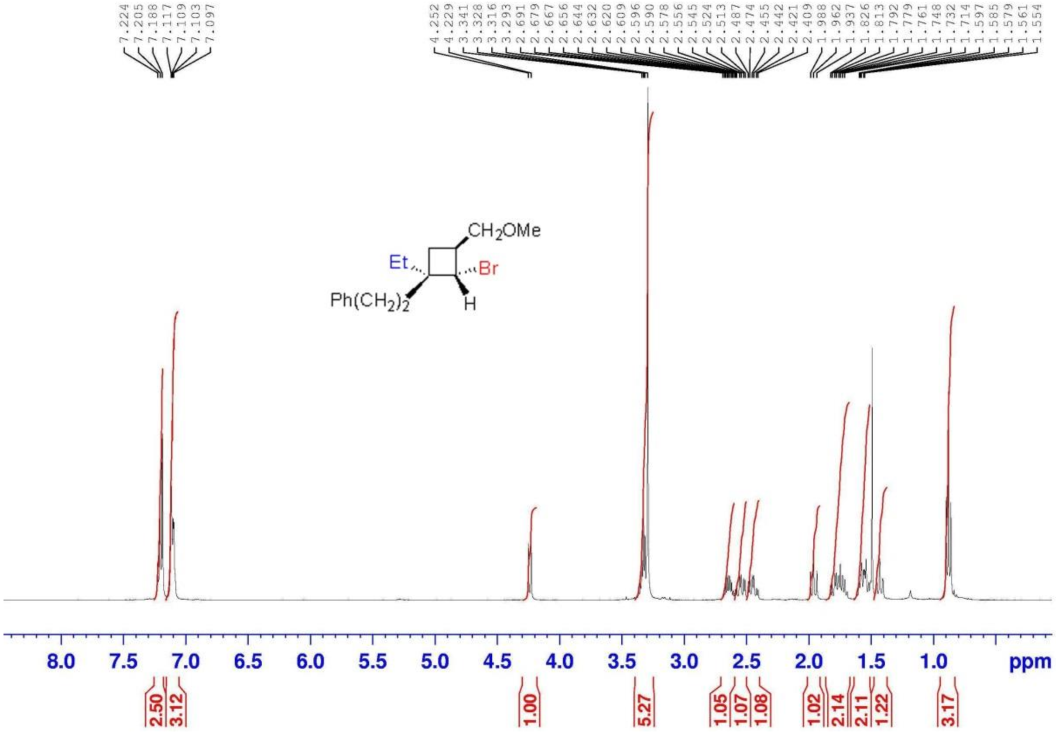

^{13}C NMR spectrum of 2e (100 MHz, $CDCl_3$):

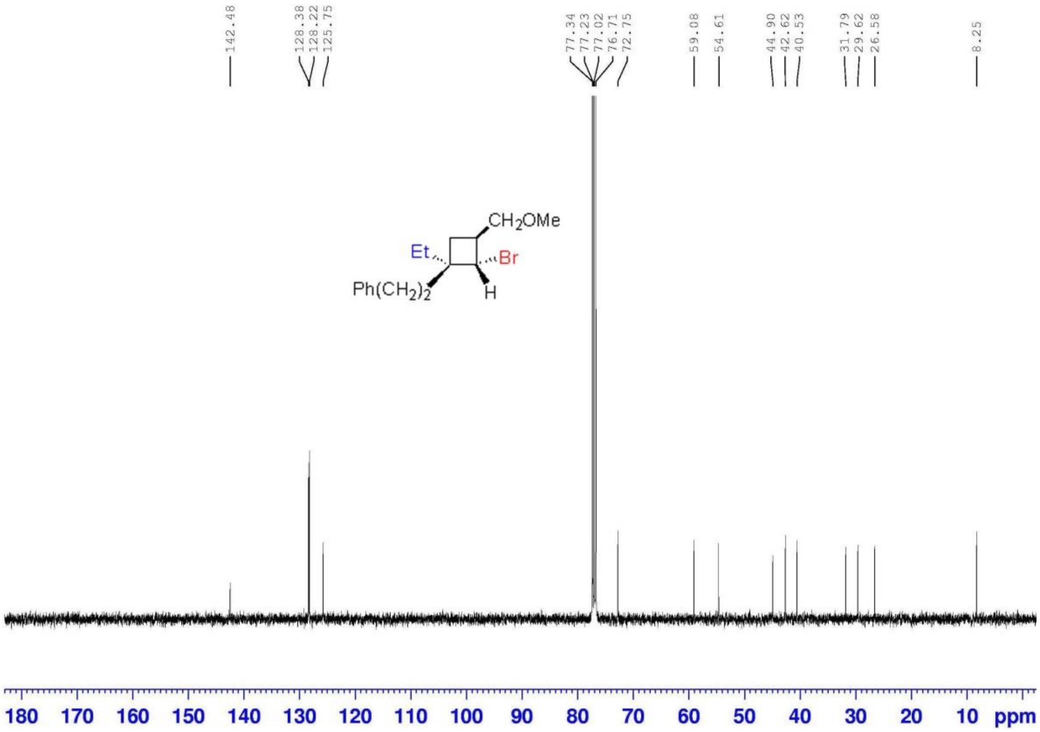

^{1}H NMR spectrum of 2f (400 MHz, $CDCl_3$):

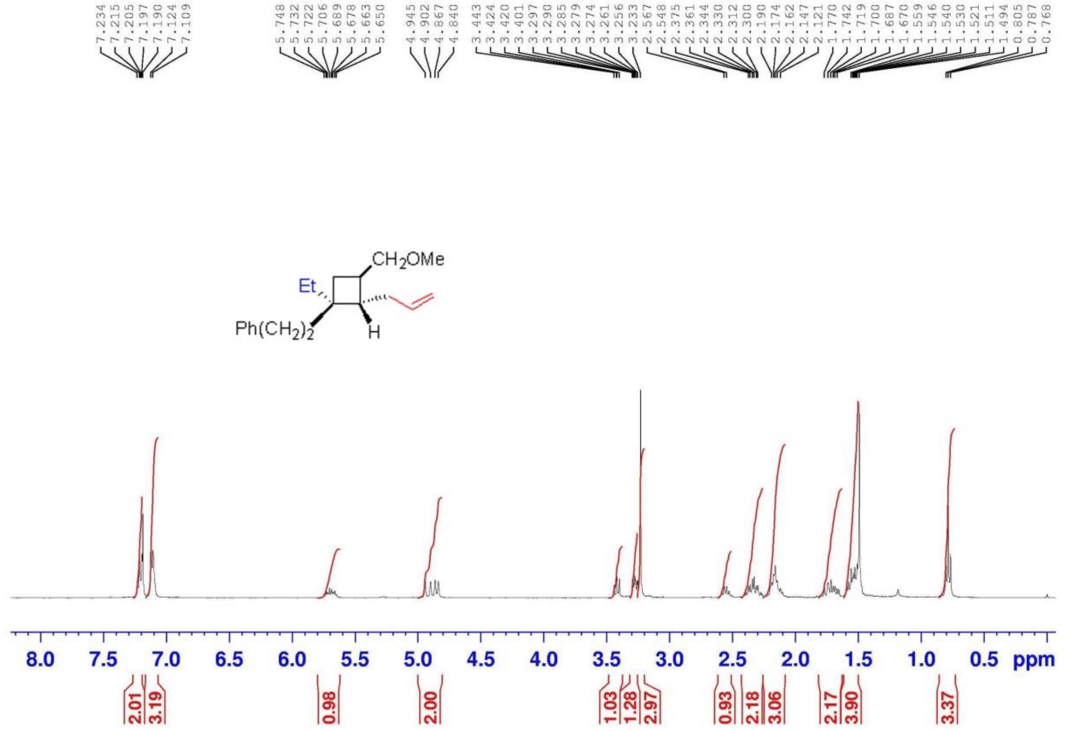

^{13}C NMR spectrum of 2f (100 MHz, $CDCl_3$):

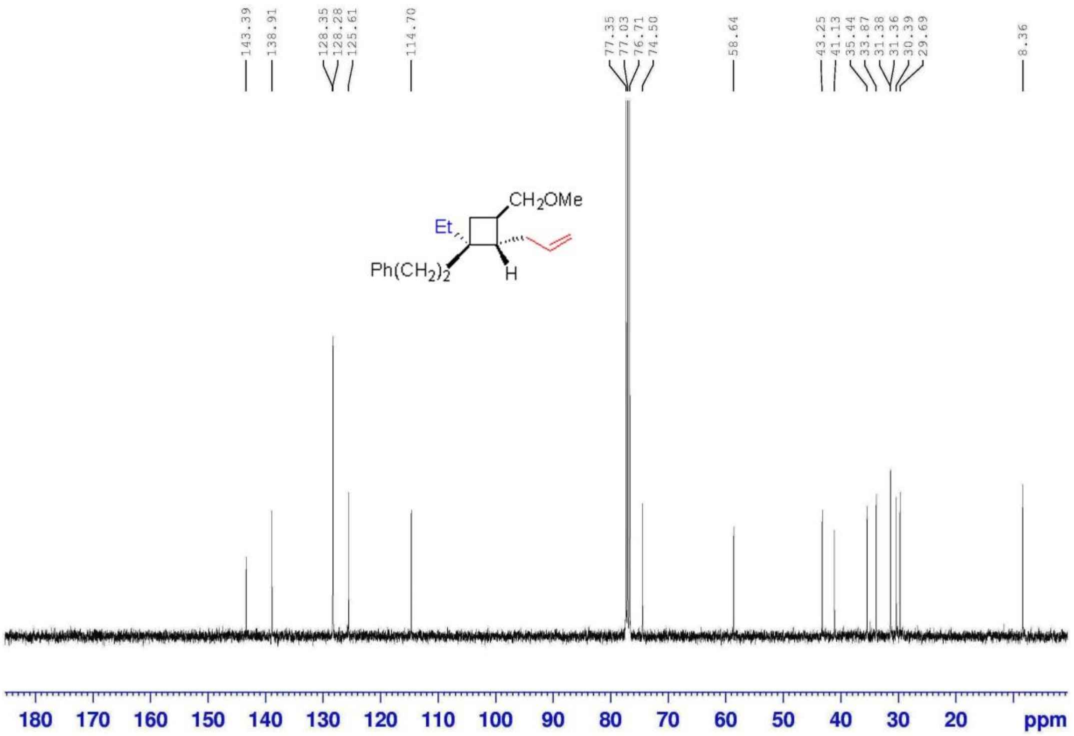

^{1}H-^{1}H NOE spectrum of 2f (600 MHz, $CDCl_3$):

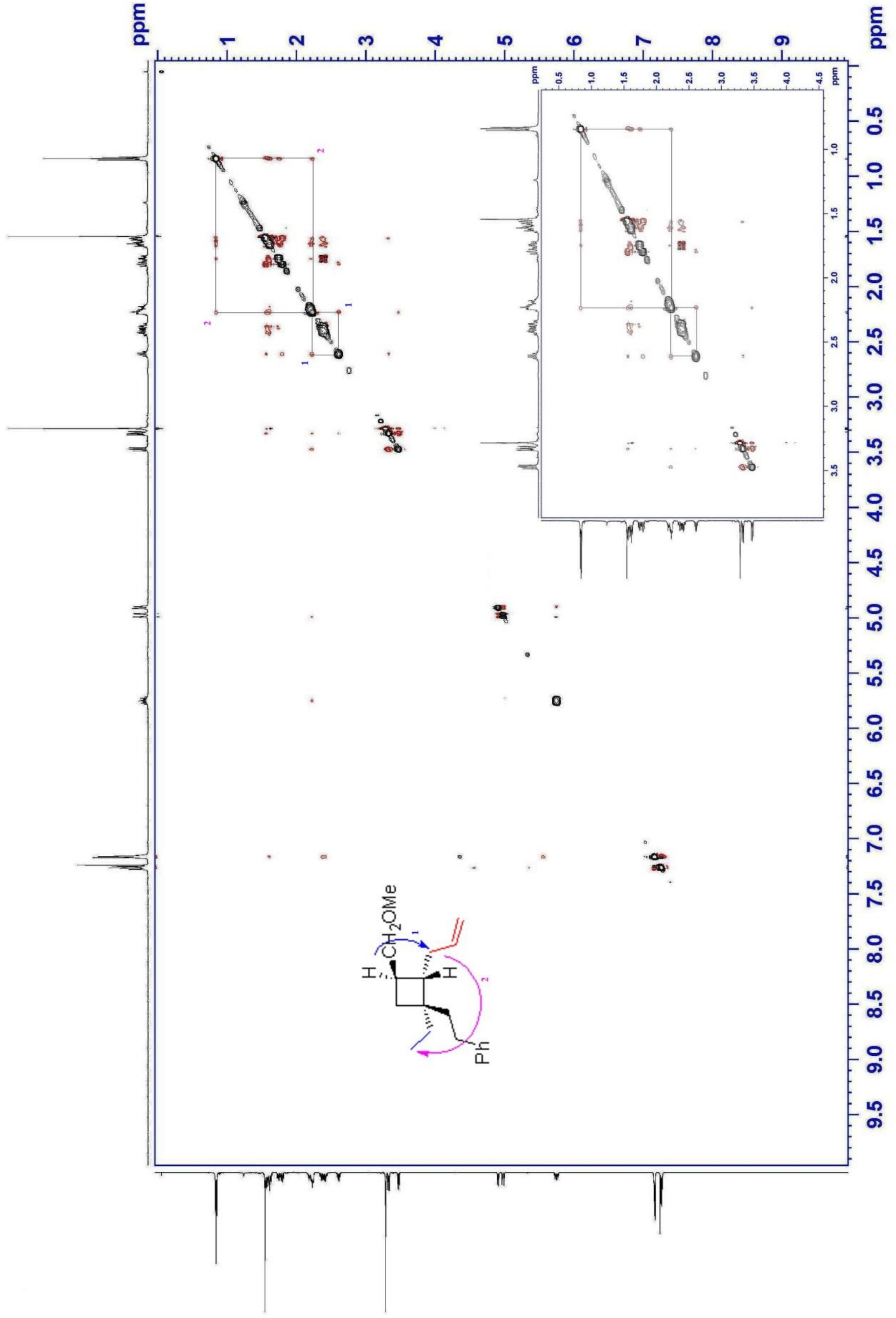

^{1}H NMR spectrum of 2g (400 MHz, $CDCl_3$):

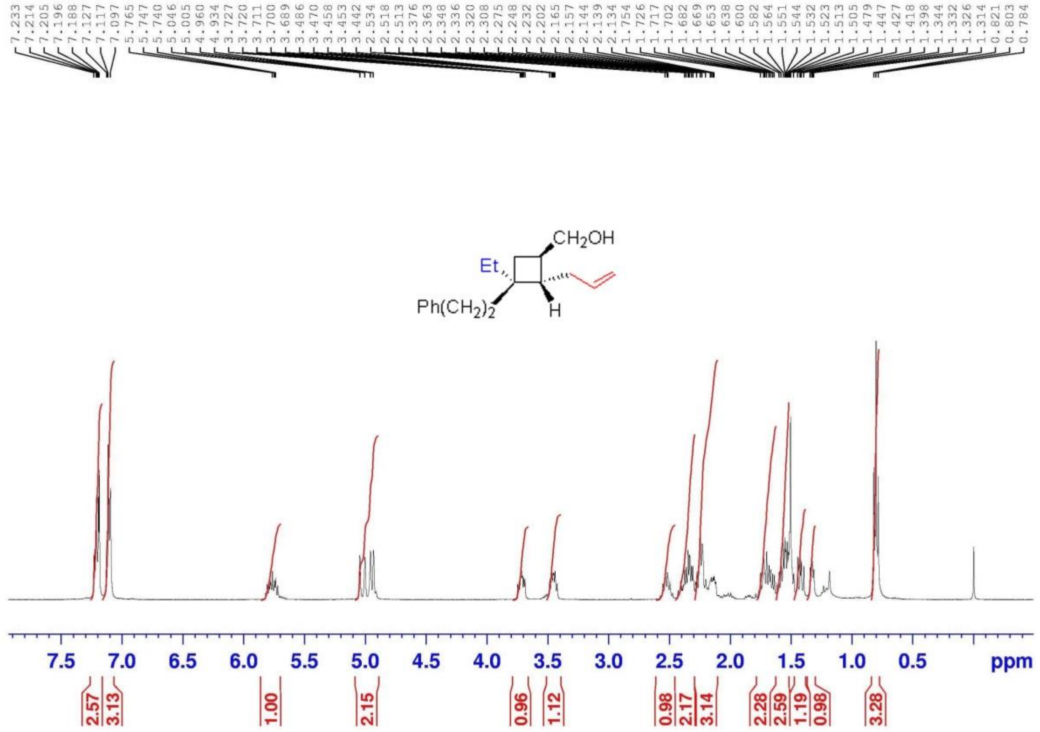

^{13}C NMR spectrum of 2g (100 MHz, $CDCl_3$):

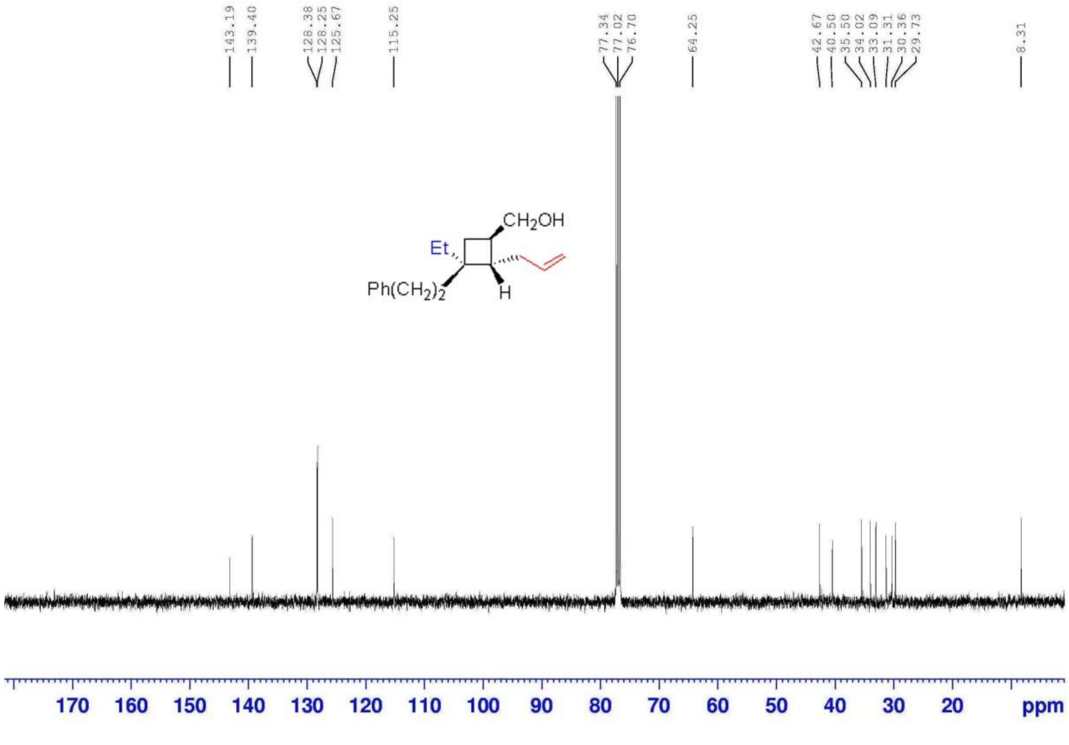

^{1}H NMR spectrum of 2h (400 MHz, $CDCl_3$):

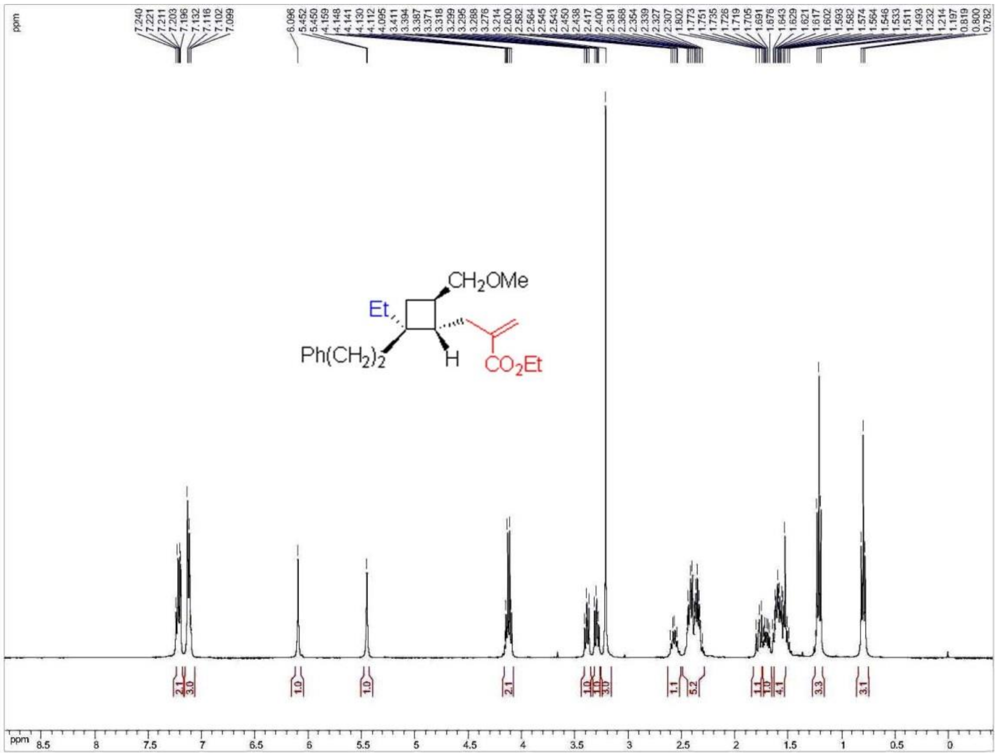

^{13}C NMR spectrum of 2h (100 MHz, $CDCl_3$):

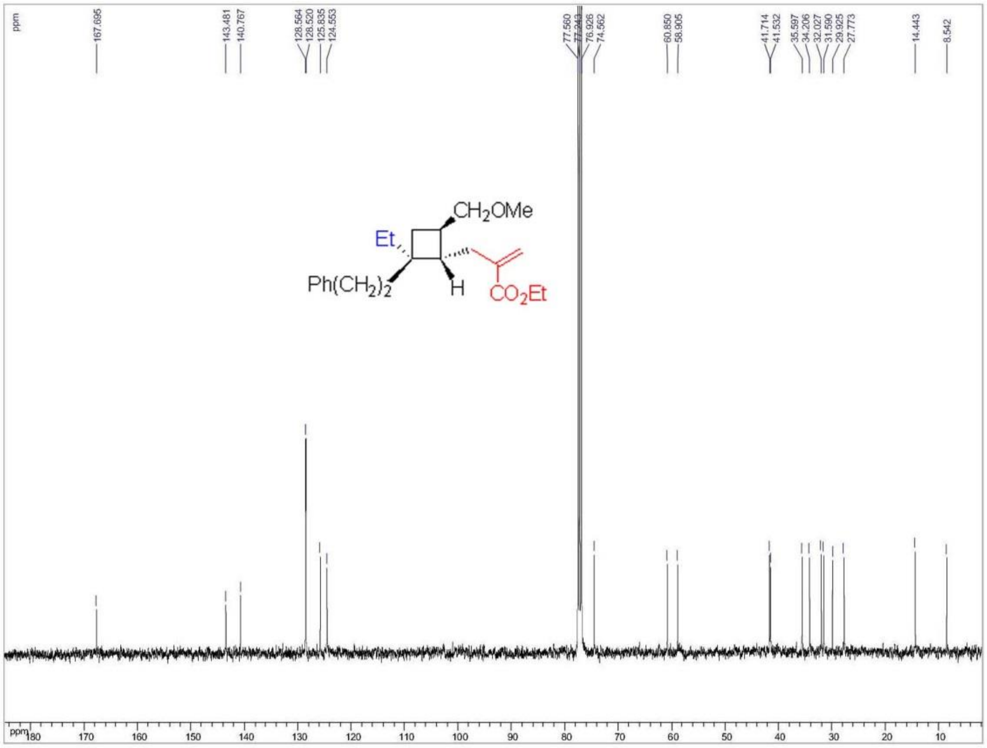

^{1}H NMR spectrum of 2i (400 MHz, $CDCl_3$):

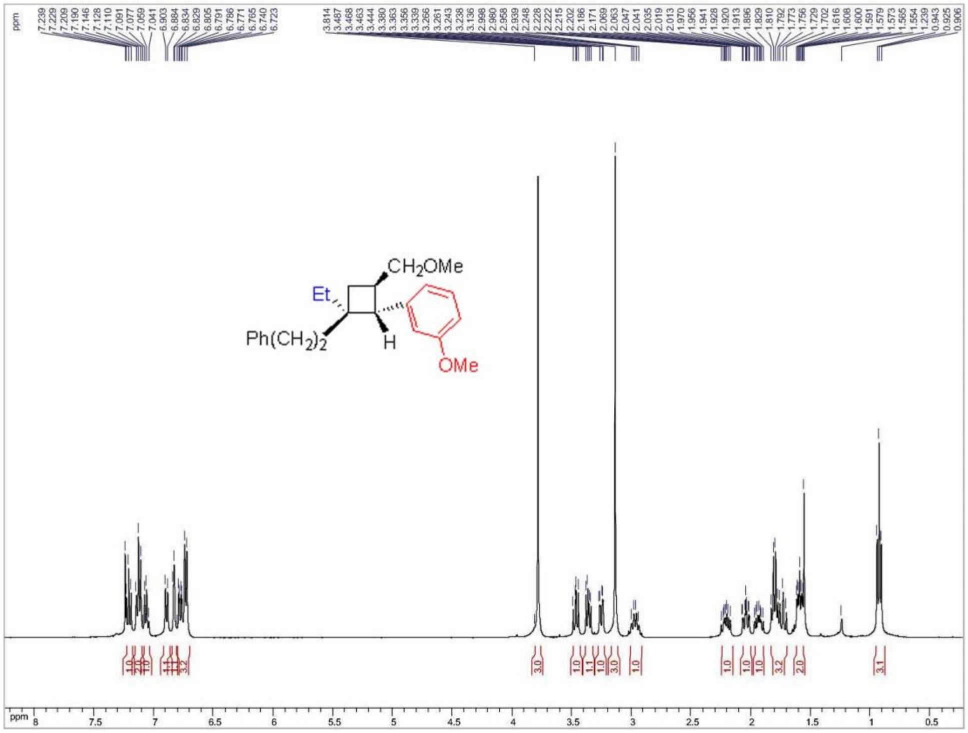

^{13}C NMR spectrum of 2i (100 MHz, $CDCl_3$):

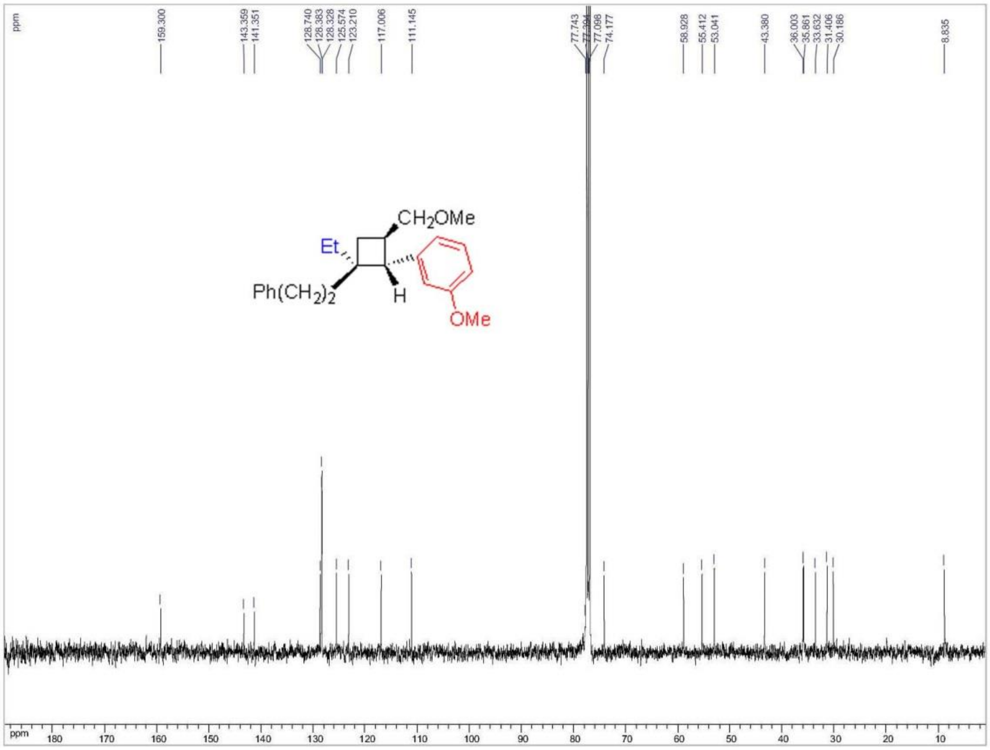

^{1}H NMR spectrum of 2j (400 MHz, $CDCl_3$):

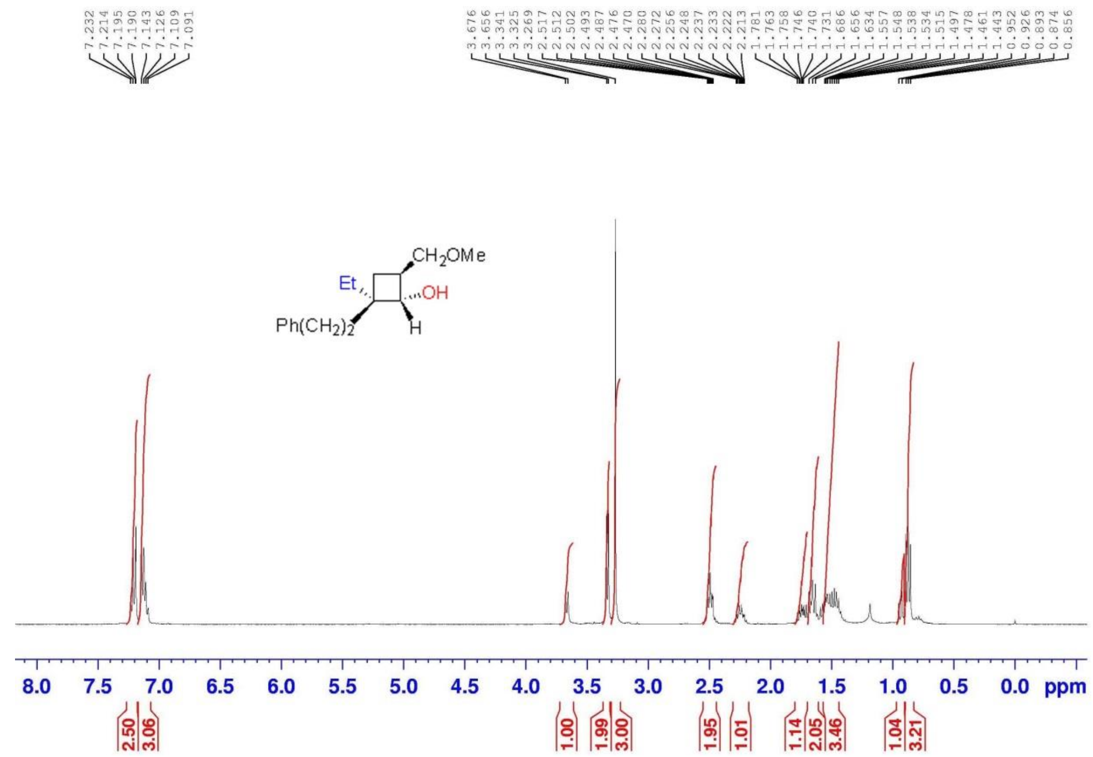

^{13}C NMR spectrum of 2j (100 MHz, $CDCl_3$):

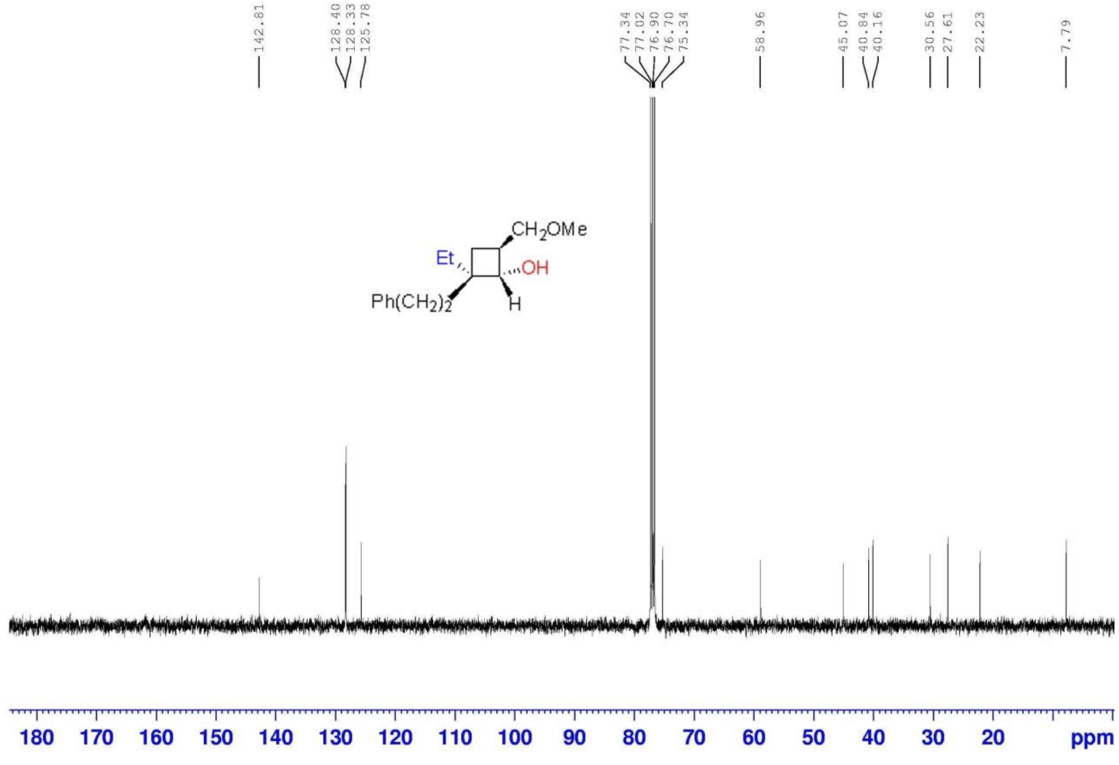

^{1}H-^{1}H NOE spectrum of 2j (600 MHz, $CDCl_3$):

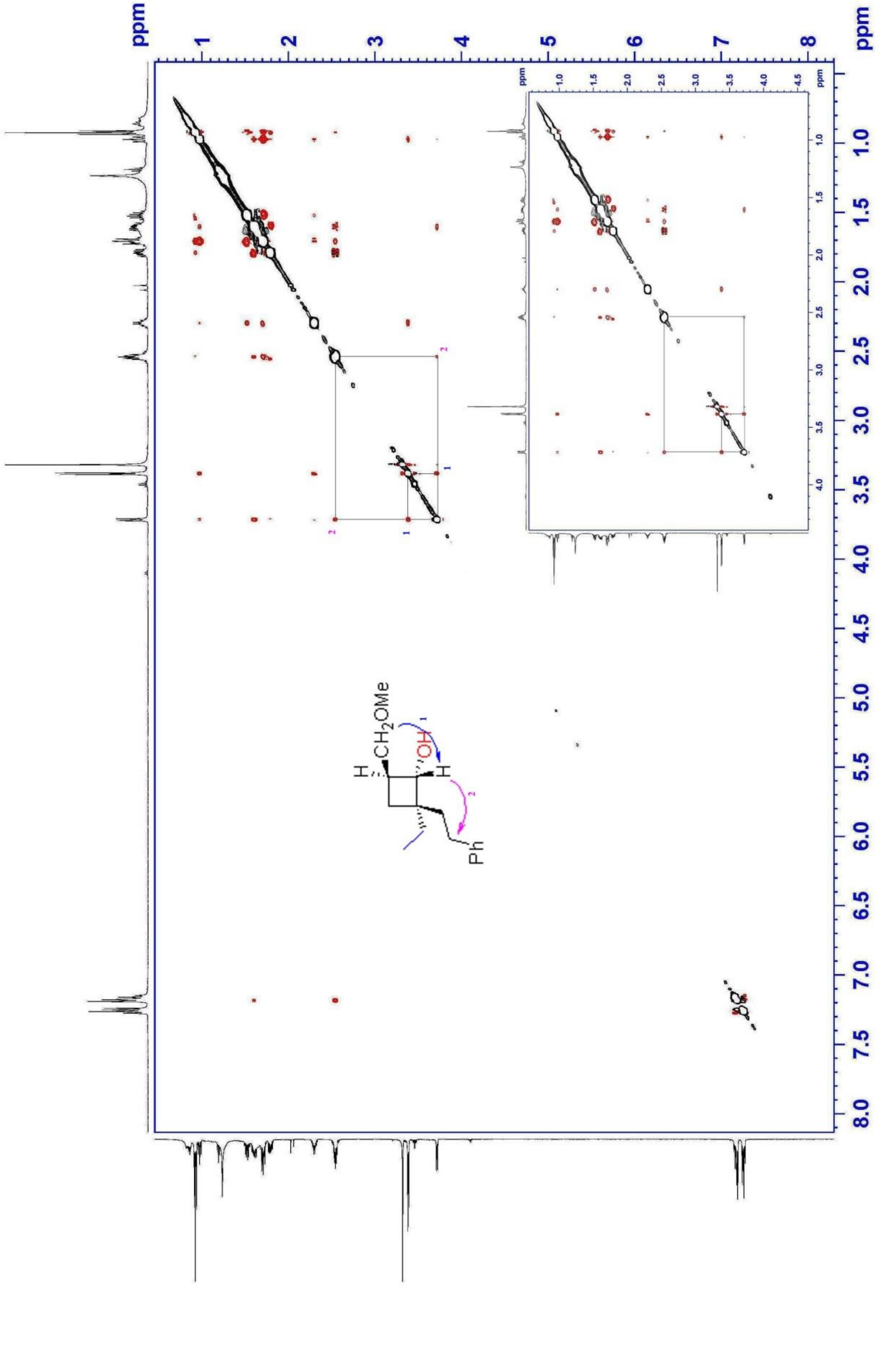

^{1}H NMR spectrum of 2k (400 MHz, $CDCl_3$):

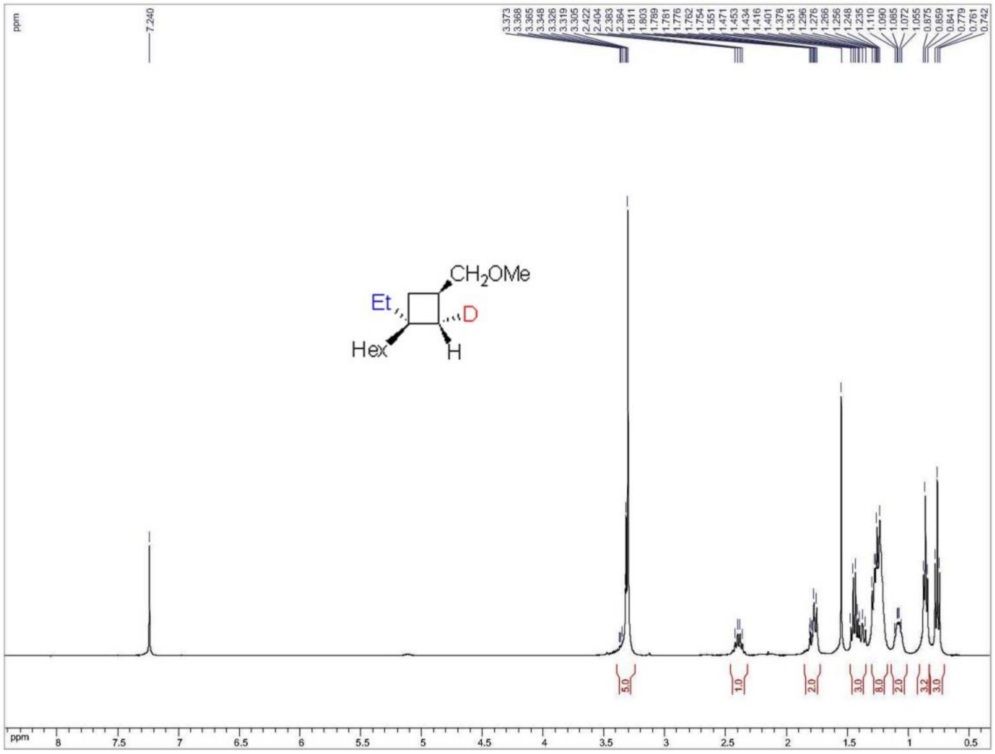

^{13}C NMR spectrum of 2k (100 MHz, $CDCl_3$):

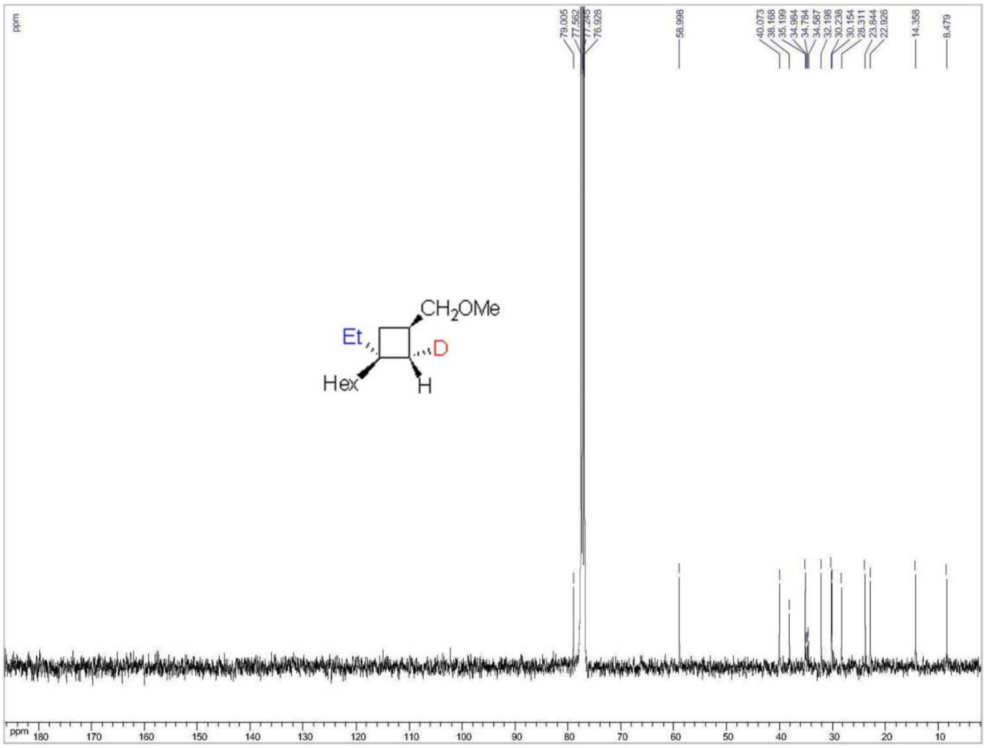

^{1}H NMR spectrum of 2l (400 MHz, $CDCl_3$):

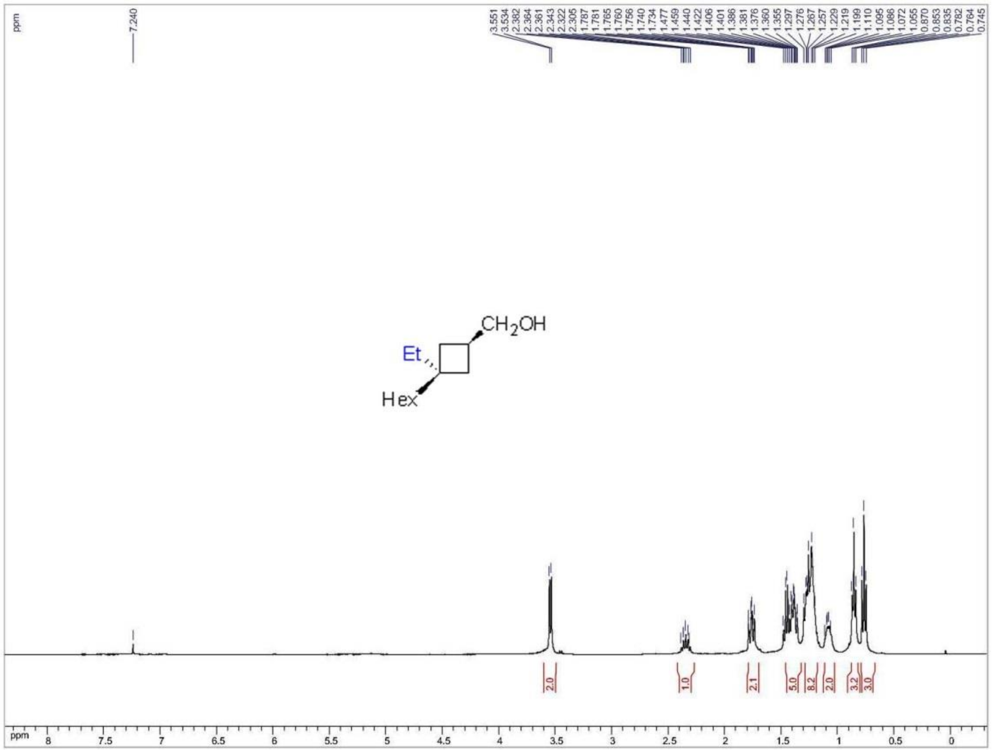

^{13}C NMR spectrum of 2l (100 MHz, $CDCl_3$):

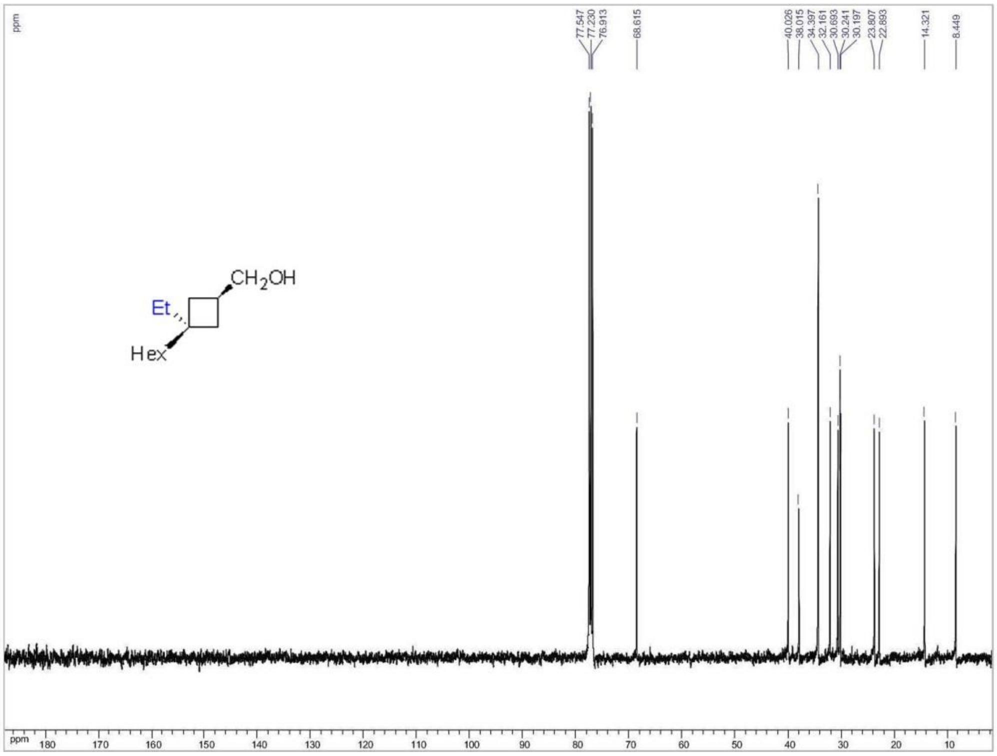

^{1}H NMR spectrum of 2m (400 MHz, $CDCl_3$):

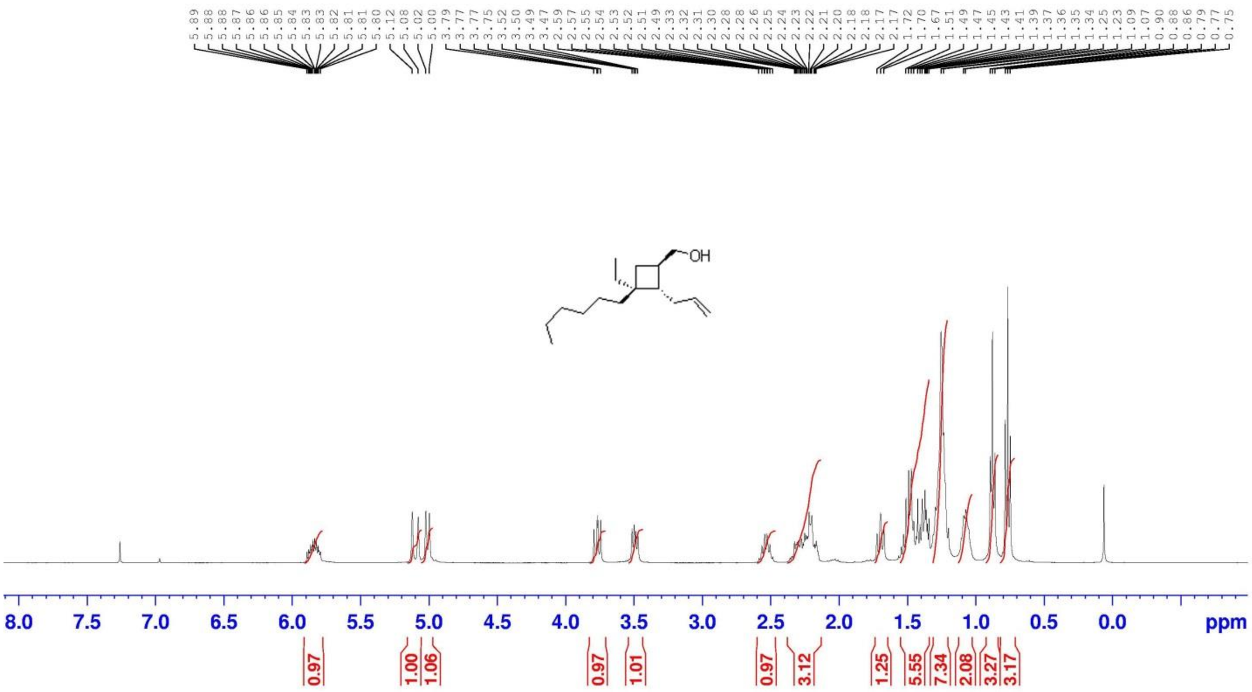

^{13}C NMR spectrum of 2m (100 MHz, $CDCl_3$):

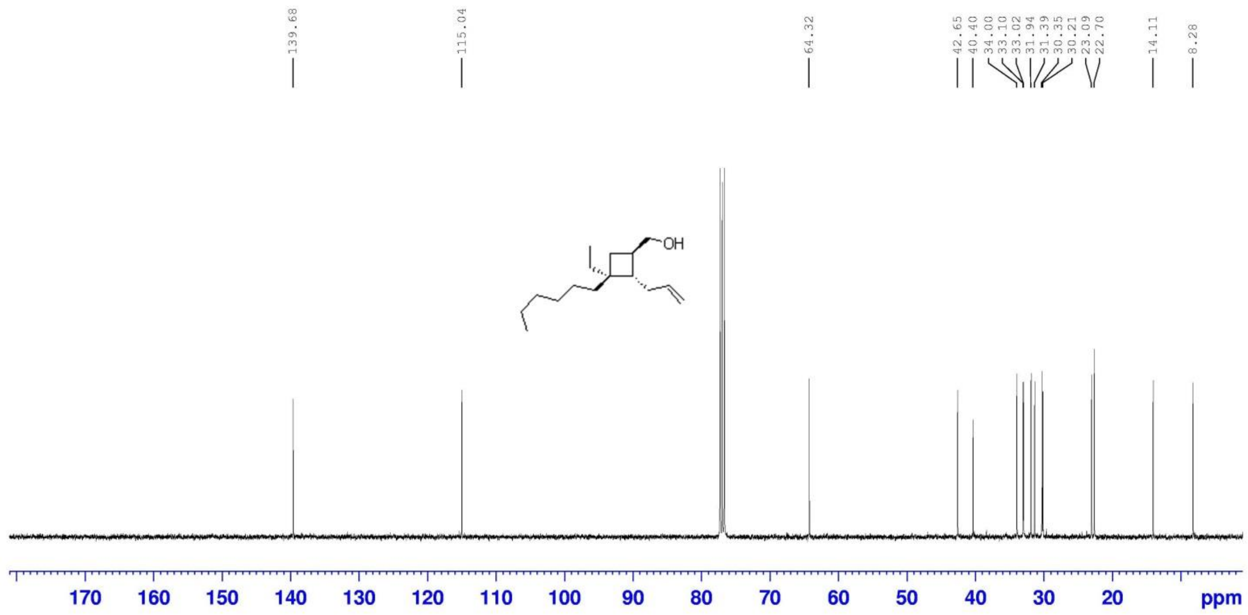

^{1}H NMR spectrum of 2n (400 MHz, $CDCl_3$):

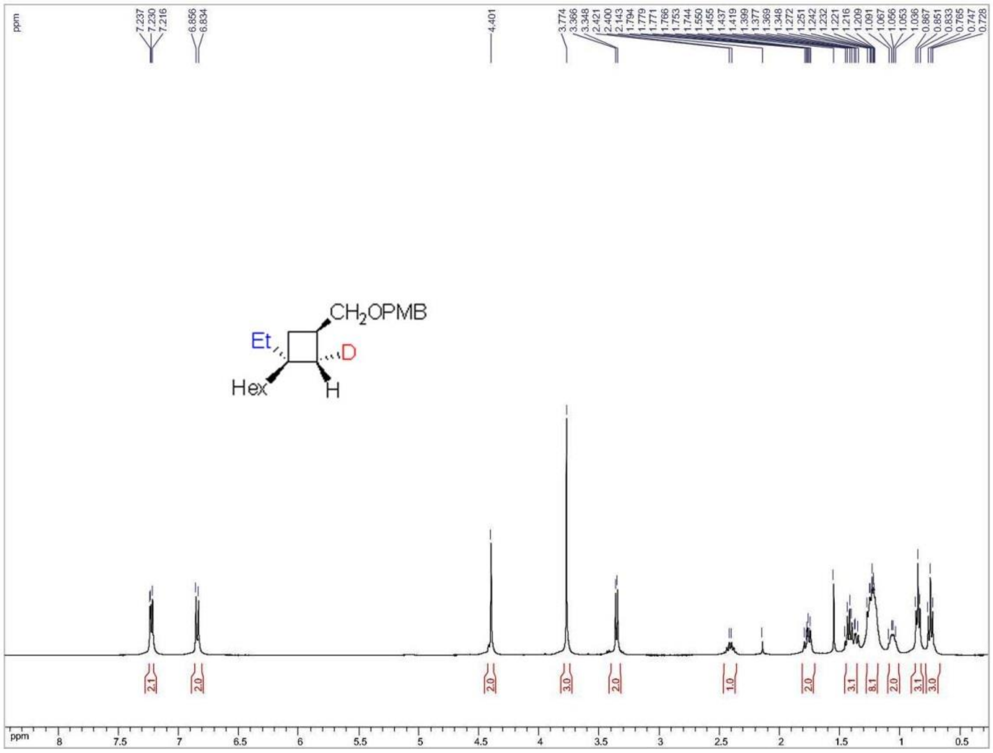

^{13}C NMR spectrum of 2n (100 MHz, $CDCl_3$):

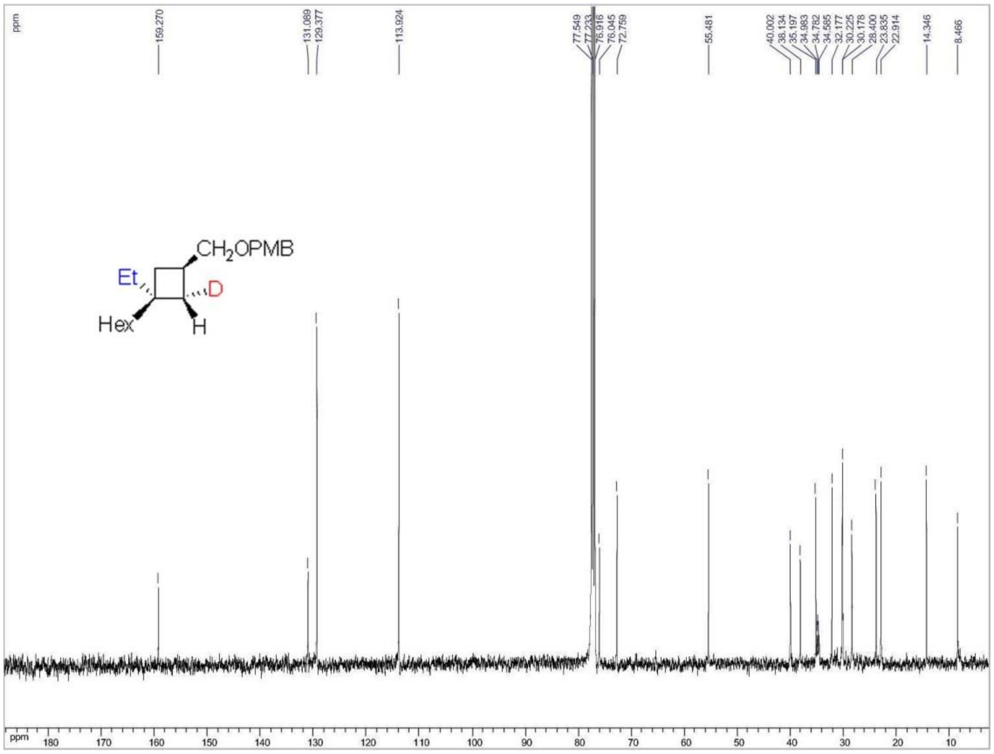

^{1}H NMR spectrum of 2o (400 MHz, $CDCl_3$):

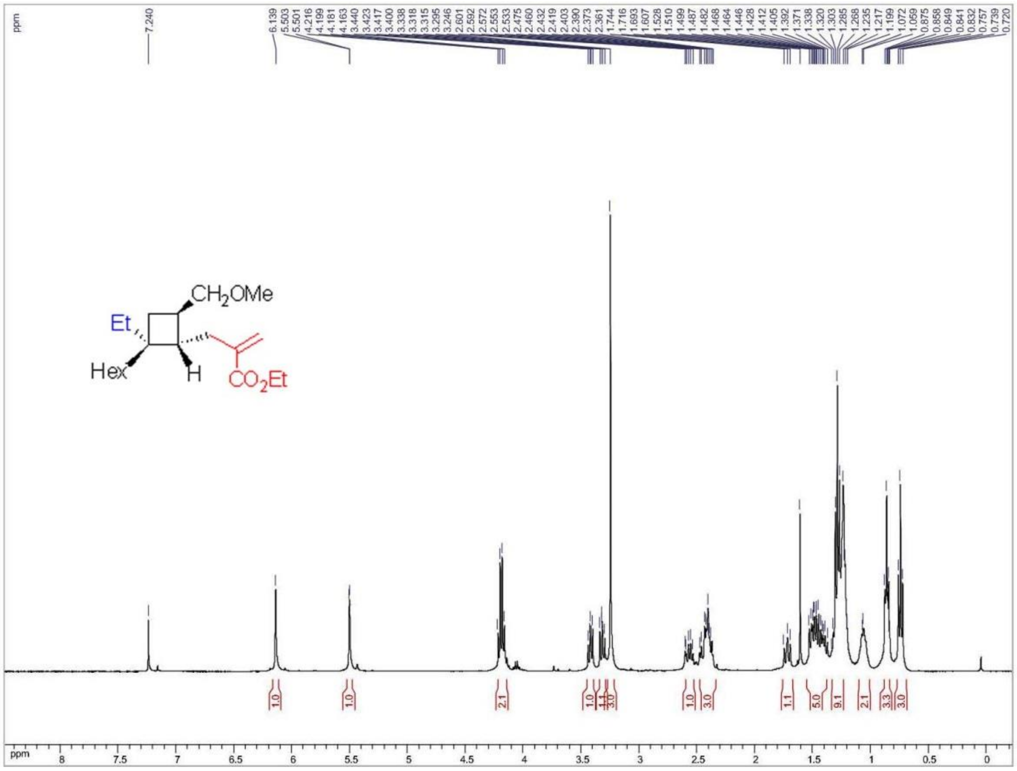

^{13}C NMR spectrum of 2o (100 MHz, $CDCl_3$):

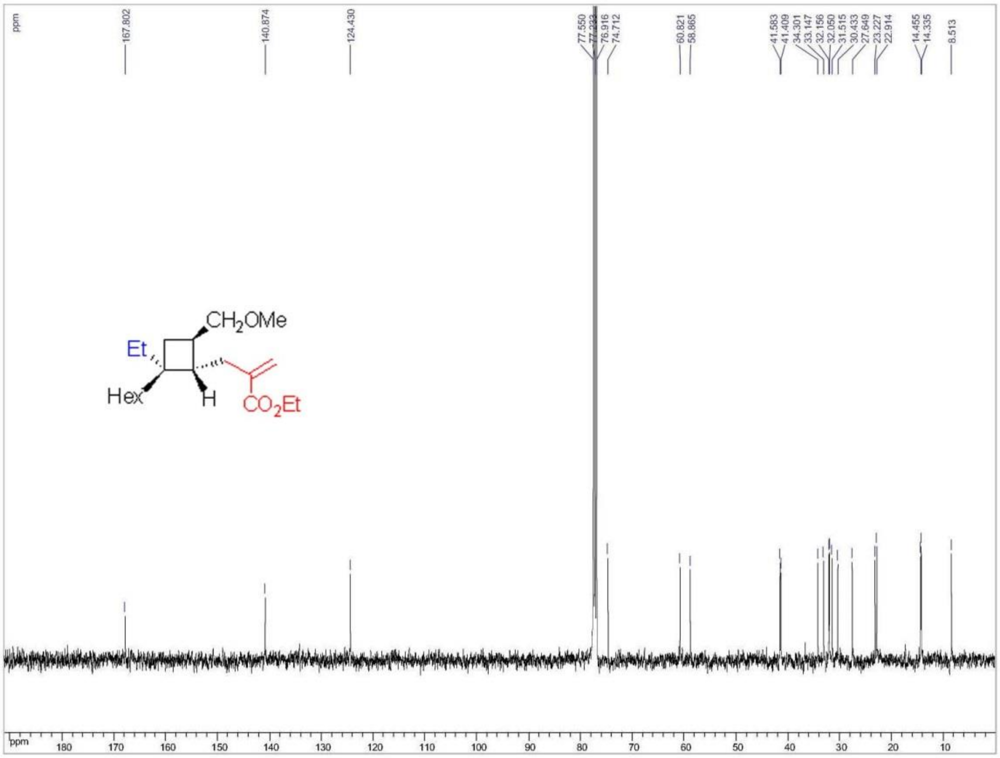

^{1}H NMR spectrum of 2p (400 MHz, $CDCl_3$):

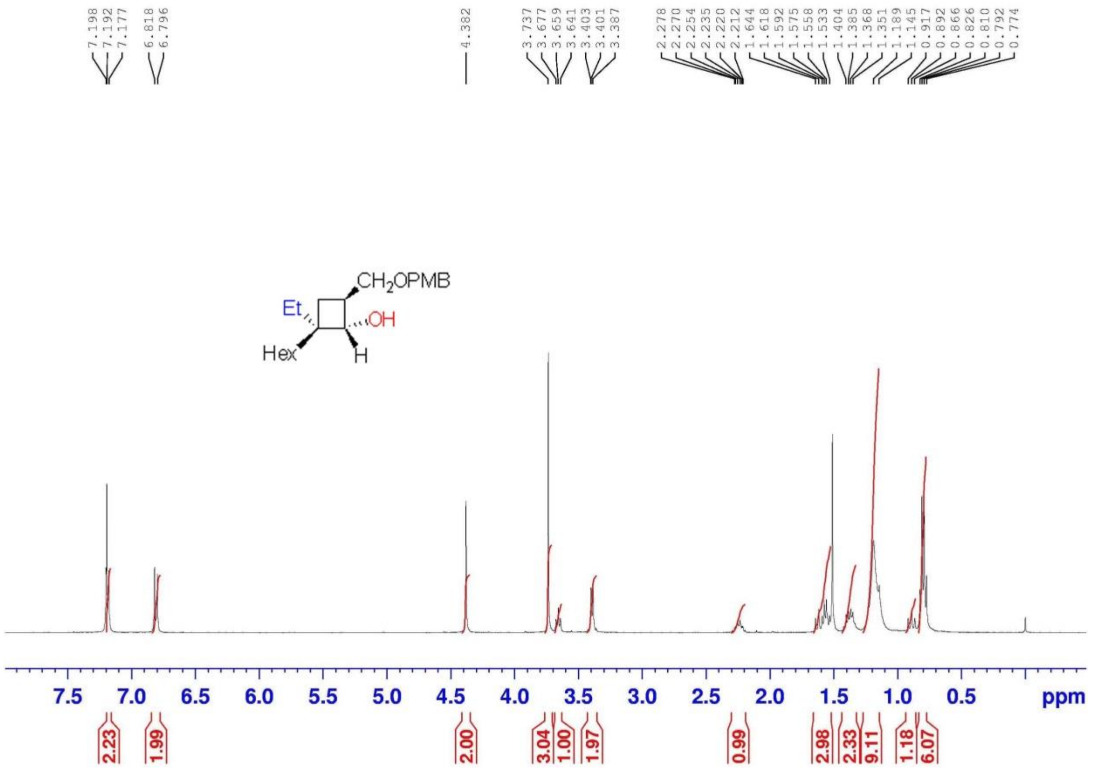

^{13}C NMR spectrum of 2p (100 MHz, $CDCl_3$):

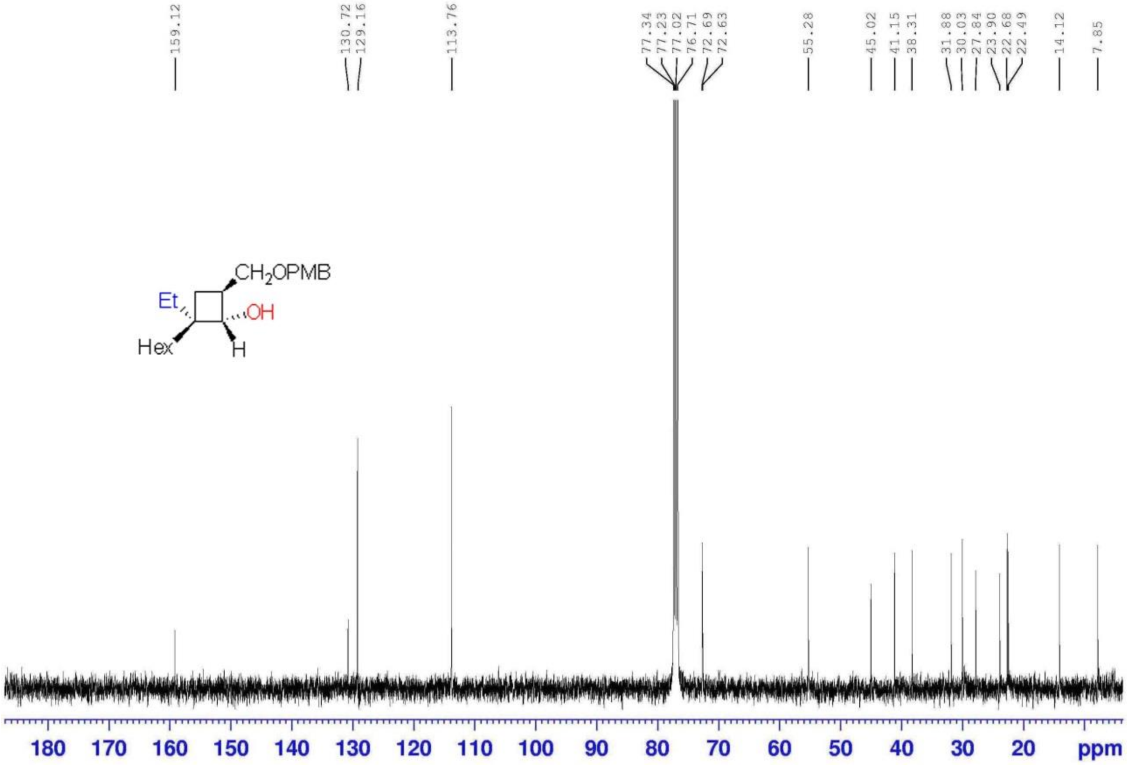

^{1}H NMR spectrum of 2q (400 MHz, $CDCl_3$):

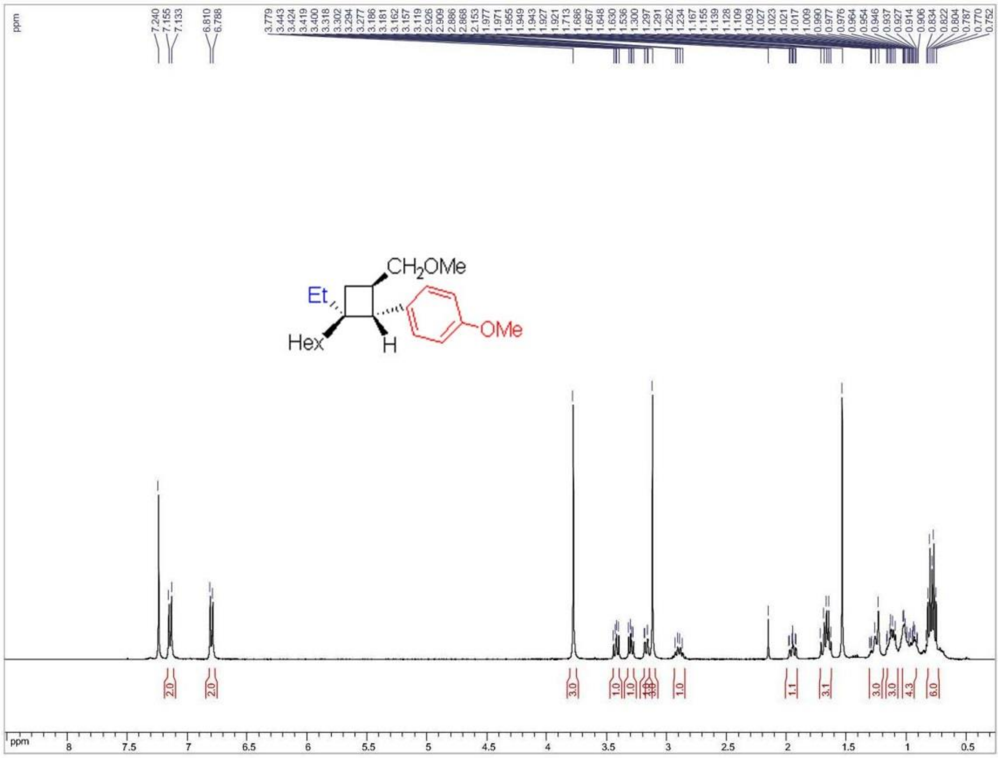

^{13}C NMR spectrum of 2q (100 MHz, $CDCl_3$):

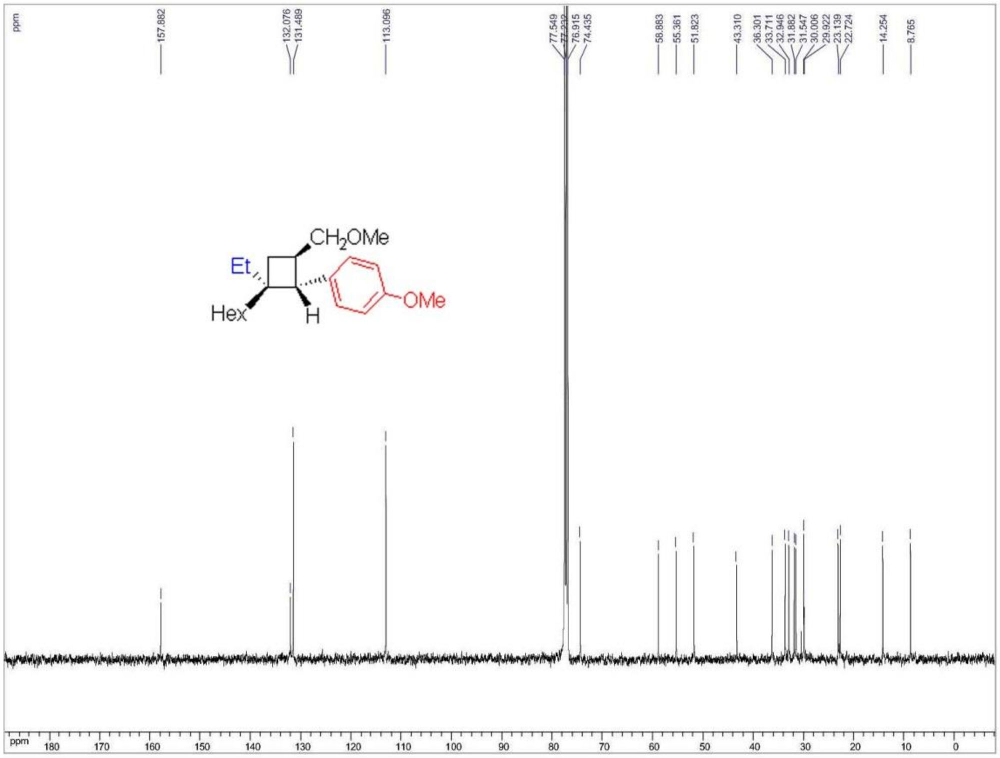

Supplement: Supplementary file 1 [file SC-008-C6SC02617F-s001.pdf]
